# Common clonal origin of conventional T cells and induced regulatory T cells in breast cancer patients

**DOI:** 10.1038/s41467-021-21297-y

**Published:** 2021-02-18

**Authors:** Maria Xydia, Raheleh Rahbari, Eliana Ruggiero, Iain Macaulay, Maxime Tarabichi, Robert Lohmayer, Stefan Wilkening, Tillmann Michels, Daniel Brown, Sebastiaan Vanuytven, Svetlana Mastitskaya, Sean Laidlaw, Niels Grabe, Maria Pritsch, Raffaele Fronza, Klaus Hexel, Steffen Schmitt, Michael Müller-Steinhardt, Niels Halama, Christoph Domschke, Manfred Schmidt, Christof von Kalle, Florian Schütz, Thierry Voet, Philipp Beckhove

**Affiliations:** 1 RCI Regensburg Centre for Interventional Immunology, University and Department of Hematology/Oncology, University Medical Centre of Regensburg, Regensburg, Germany; 2 Translational Immunology Department, German Cancer Research Centre, Heidelberg, Germany; 3 The Cancer, Ageing and Somatic Mutation Program, Wellcome Sanger Institute, Hinxton, UK; 4 Translational Oncology Department, National Centre for Tumor Diseases and German Cancer Research Centre, Heidelberg, Germany; 5 Technical Development, Earlham Institute, Norwich, UK; 6 The Francis Crick Institute, London, UK; 7 Institute for Theoretical Physics, University of Regensburg, Regensburg, Germany; 8 Department of Human Genetics, University of Leuven, KU Leuven, Leuven, Belgium; 9 Medical Oncology Department, National Centre for Tumor Diseases, Heidelberg, Germany; 10 Hamamatsu Tissue Imaging and Analysis Centre, BIOQUANT, University of Heidelberg, Heidelberg, Germany; 11 Flow Cytometry Core Facility, German Cancer Research Centre, Heidelberg, Germany; 12 German Red Cross (DRK Blood Donation Service in Baden-Württemberg-Hessen) and Institute for Transfusion Medicine and Immunology, Medical Faculty Mannheim, Heidelberg University, Mannheim, Germany; 13 Department of Gynecology and Obstetrics, University Hospital of Heidelberg, Heidelberg, Germany; 14 Present Address: The Walter and Eliza Hall Institute of Medical Research, Melbourne, VIC Australia; 15 Present Address: Centre for Cardiovascular and Metabolic Neuroscience, Department of Neuroscience, Physiology and Pharmacology, University College London, London, UK; 16 Present Address: Clinical Study Centre, Charité/BIH, Berlin, Germany

**Keywords:** Immunosurveillance, Immune evasion, Regulatory T cells, T-helper 1 cells, T-cell receptor

## Abstract

Regulatory CD4^+^ T cells (Treg) prevent tumor clearance by conventional T cells (Tconv) comprising a major obstacle of cancer immune-surveillance. Hitherto, the mechanisms of Treg repertoire formation in human cancers remain largely unclear. Here, we analyze Treg clonal origin in breast cancer patients using T-Cell Receptor and single-cell transcriptome sequencing. While Treg in peripheral blood and breast tumors are clonally distinct, Tconv clones, including tumor-antigen reactive effectors (Teff), are detected in both compartments. Tumor-infiltrating CD4^+^ cells accumulate into distinct transcriptome clusters, including early activated Tconv, uncommitted Teff, Th1 Teff, suppressive Treg and pro-tumorigenic Treg. Trajectory analysis suggests early activated Tconv differentiation either into Th1 Teff or into suppressive and pro-tumorigenic Treg. Importantly, Tconv, activated Tconv and Treg share highly-expanded clones contributing up to 65% of intratumoral Treg. Here we show that Treg in human breast cancer may considerably stem from antigen-experienced Tconv converting into secondary induced Treg through intratumoral activation.

## Abstract (web summary)

The mechanisms that shape the regulatory T cell repertoire in patients with cancer are not completely understood. Here, the authors observe that, in breast cancer patients, tumor-resident regulatory T cells do not show clonal relationship with their circulating counterpart, but share a common origin with intratumoral antigen-experienced conventional T cells.

## Introduction

Treg constitute a CD4^+^ T-cell (TC) subset identified by the surface expression of IL-2 receptor alpha chain (IL2RA, also known as CD25)^1^ and the intranuclear expression of transcription factor forkhead box P3 (FOXP3)^2^. Upon antigen-specific T-cell receptor (TCR) stimulation^1^, classical suppressive Treg inhibit Tconv function^1^, and downmodulate immune responses against both self and foreign antigens through various mechanisms^3^. These include the release of the immune-suppressive cytokines IL10, TGFB, and IL35, Teff cytolysis through perforin and granzymes, metabolic disruption through IL-2 consumption, CD39-mediated adenosine release but also inhibition of dendritic cell maturation and function through CTLA4 and LAG3^3^. Thus, Treg are powerful mediators of immune homeostasis, while protecting from autoimmunity and immunopathology^1^. On the other hand, their suppressive properties are detrimental to cancer immunity. Recognizing the tumor as self, Treg, particularly tumor-infiltrating subsets, not only inhibit tumor eradication by tumor-antigen (TA)-reactive Teff^4^ through classical suppressive mechanisms but also exert direct nonimmune pro-tumorigenic effects through the production of molecules that support cancer development^5^. Among these are VEGF, which promotes enhanced tumor angiogenesis^6^, AREG, which induces lung tumor growth^7^, and RANKL, which drives lung metastasis of breast cancer^8^. While Treg enrichment in tumors and peripheral blood (PB) of patients with cancer^9^, including breast cancer^10^, associates with impaired anti-tumor immune responses and poor survival^9,11,12^, Treg depletion in mice restores cancer immunity, inducing tumor clearance^9^. Consequently, the balance between induction, expansion, and tumor-infiltration of TA-specific Teff and TA-specific Treg is a critical determinant of tumor progression. Thus, understanding the mechanisms of Treg repertoire formation in tumor patients is of major importance for the development of efficient immunotherapeutic strategies against cancer.

While originally Treg development was only assigned to the thymus (natural Treg; nTreg), recent data demonstrate that Treg can also be generated in the periphery (outside the thymus) from originally FOXP3^−^ Tconv under suboptimal TCR stimulation conditions (peripherally induced Treg; pTreg or iTreg)^13–15^. iTreg may develop not only from antigen-inexperienced naïve Tconv (primary iTreg) but also from antigen-experienced Teff/memory clones upon secondary antigen encounter (secondary iTreg), as supported by studies in lymphopenic mice and in vitro experiments exposing Teff and memory TC to TGFB upon TCR stimulation^16,17^. To date, antigen-specific cancer immunotherapies (e.g., vaccination, adoptive T-cell therapy) aim at increasing the numbers of TA-specific Teff within the patient’s tumor, although, so far, it remains elusive whether antigen-experienced TA-specific Teff may acquire an adverse functional suppressive Treg phenotype instead of executing anti-tumor activity in cancer patients. This may not be unlikely since tumors produce suppressive factors, including TGFB, that support both proliferation of pre-existing nTreg and conversion of Tconv into iTreg^9^. Most importantly, in tumor-bearing mice, iTreg can contribute not only to the peripheral Treg pool but also to tumor-specific immune tolerance within the tumor tissue^15,18^. TC recognize their specific antigen through the TCR, which is generated in the thymus through V(D)J recombination. Although this process is biased towards particular V–J gene segment combinations^19,20^, the possibility of bias toward a specific CDR3 nucleotide sequence within the same individual is extremely rare^21,22^. While identical TCR nucleotide sequences detected in more than one cell indicate clonal expansion following antigen encounter^23,24^ and, therefore, identify antigen-experienced TC clones, overlapping TCR sequences between Treg and Tconv indicate conversion of antigen-experienced Tconv into secondary iTreg. Using TCR sequencing a recent study in the MCA (3-methylcholanthrene)-chemically induced sarcoma tumor mouse model revealed no TCR overlap between antigen-experienced Tconv and Treg, arguing against Treg conversion from expanded Tconv clones in vivo^15^.

In humans, naive Tconv convert into suppressive iTreg in vitro upon polyclonal stimulation and exposure to TGFB^25^. However, whether iTreg are generated in vivo in humans at all remains open. Although single-cell studies in colorectal carcinoma patients have shown TCRαβ overlap between FOXP3^−^ and FOXP3^+^ TC within the tumor^26,27^, their Tconv or suppressive Treg functional identity was not conclusively determined. Moreover, in human colorectal cancer, several reports of a positive correlation between increased FOXP3^+^ Treg tumor-infiltration and favorable prognosis^28^ suggest that particularly in this cancer entity FOXP3^+^ TC may not correspond to suppressive Treg but rather to ActTconv that transiently upregulate FOXP3 upon antigen recognition^29^. In breast cancer patients bulk TCR repertoire analysis revealed overall low similarity between tumor-derived Tconv and Treg^30,31^, arguing against a major contribution of secondary iTreg to Treg enrichment in tumor patients. However, the former study utilized algorithm-based TCRβ extraction from sequencing data of total RNA amplification rather than quantitatively unbiased TCR-specific PCR products^30^, while the latter compared bulk sequences of enriched Treg and Tconv populations from five pooled patients^31^. Thus, both studies may report clonal frequency distribution and, consequently, TCR similarities that do not precisely reflect the ones in the individual tumors. Recently, Su et al.^31^ showed conversion of naïve Tconv into suppressive primary iTreg in vitro upon exposure to APCs and cancer cell supernatants from autologous breast tumors, suggesting that intratumoral Treg may derive from naive Tconv.

In this work, we investigate Treg repertoire formation in breast cancer patients using bulk and single-cell TCR sequencing combined with single-cell transcriptome sequencing of Treg and Tconv CD4^+^ TC from peripheral blood and breast tumors. Here we show that the intratumoral suppressive Treg repertoire is distinct from the circulating Treg population but has a common clonal origin with tumor-infiltrating antigen-experienced Tconv, suggesting intratumoral Tconv conversion into secondary iTreg through an intermediate activated phenotype.

## Results

### Oligoclonal expansion of TA-reactive Teff in patients’ blood

**Fig. 1:**
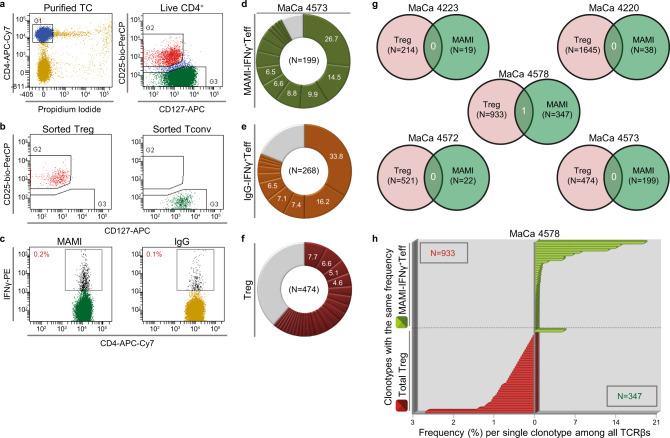
Minor TCR similarity between TAA-reactive Teff and total Treg in PB of breast cancer patients. **a** Separation of live CD4^+^ TC (G1, blue) into CD25^+^CD127^-/low^ (G2, red) total Treg and CD25^−^CD127^+/−^ (G3, green) total Tconv using FACS sorting. G, Gate. **b** FACS re-analysis of sorted Treg and Tconv. **c** Frequency of IFNγ^+^Teff among total Tconv after stimulation with MAMI (TAA) or IgG (negative control) based on IFNγ-catch assay and FACS analysis. **d**–**f** Frequency distribution of all clonotypes (unique TCRβ nucleotide sequences) obtained from (**d**) MAMI-IFNγ^+^Teff, (**e**) IgG-IFNγ^+^Teff, and (**f**) total Treg presented in a clockwise pie chart, one colored pie-slice per clonotype, in order of decreasing percentage (%, white). Gray slices correspond to the TCRβ repertoire fraction that contains clonotypes with a frequency below 1%. *N*, total number of clonotypes. A representative example from 1 out of (**a**) *n* = 11, (**b**) *n* = 6, (**c**, **d**, **f**) *n* = 5, (**e**) *n* = 3 biologically independent replicates. **g** Venn diagram showing the number of common (white) clonotypes between MAMI-IFNγ^+^Teff and total Treg per breast cancer patient with mammary gland adenocarcinoma (MaCa). **h** Frequency of common clonotypes shared between MAMI-IFNγ^+^Teff and total Treg in relation to the frequencies of all clonotypes detected per subset. Due to the big number of identified clonotypes per subset, each bar represents a group of clonotypes with identical frequencies (*y* axis) plotted against the frequency of each clonotype in this group (*x* axis). Clonotypes are shown in order of decreasing (Treg, red) or increasing (MAMI-IFNγ^+^Teff, green) frequency and the clonotypes of each subset are separated by a dashed black line. In case of an overlapping clonotype between the two subsets, a red and a green bar align on the *y* axis. *N*, total number of clonotypes identified among Treg (red) and MAMI-IFNγ^+^Teff (green). **g**, **h** *n* = 5 biologically independent replicates. Source data are provided as a Source Data file.

IFNγ-secreting TA-reactive Teff are enriched in breast cancer patients, exhibit potent tumor-killing properties, and possess therapeutic potential^32,33^. However, due to their high frequencies and their capacity to infiltrate and recognize tumors, they represent a potentially relevant and critical source of tumor-reactive secondary iTreg. To clarify whether in human cancers antigen-experienced Teff or memory Tconv contribute to the Treg repertoire in vivo, we compared the TCR repertoires of antigen-experienced TA-specific CD4^+^Teff clones and Treg in PB of breast cancer patients. We focused on CD4^+^Teff reactive to the tumor-associated antigen (TAA) MAMI, a 50 amino acid (aa)-long peptide derived from Mammaglobin, which is commonly overexpressed in breast tumors^34^. Breast cancer patients harbor in PB IFNγ-producing CD4^+^Teff but also suppressive CD4^+^Treg reactive to MAMI^33,35,36^. Consequently, CD4^+^Treg and MAMI-responding IFNγ^+^CD4^+^Teff may share the same TCRs due to peripheral iTreg generation. To investigate this possibility, the TCR repertoire of MAMI-reactive IFNγ^+^CD4^+^Teff was compared to the TCR pool of total CD4^+^Treg in PB of five breast cancer patients with mammary gland adenocarcinoma (MaCa). To this end, we sequenced the TCRβ-chain (*TRB*; *TCRB*) transcript of each subset separately and used the CDR3 nucleotide sequence to trace individual TC clones and identify their frequency in either or both subsets. We used FACS sorting based on the surface expression of CD25 and the interleukin 7 receptor (IL7R; CD127) to separate PB-derived TC into CD4^+^CD25^+^CD127^−/low^ Treg and CD4^+^CD25^−^CD127^−/+^ Tconv^1,37^ (Fig. 1a and Supplementary Fig. 1a), as low CD127 expression correlates with a FOXP3-positive phenotype, the most reliable Treg-specific marker^2^, and suppressive function^38^. After-sorting purity of both populations was above 98% with maximum expected contamination from the other subset during sorting calculated at 3% (Fig. 1b, Supplementary Figs. 1b and 2a and Supplementary Table 1). CD25^+^FOXP3^+^ cells were not only highly enriched in sorted Treg at a proportion above 97% but were also efficiently depleted among sorted Tconv (Supplementary Fig. 2a). Thus, sorted Treg and Tconv populations were highly pure with limited contamination from the opposite subset. Subsequently, Tconv were stimulated with autologous dendritic cells (DC) presenting MAMI or human Immunoglobulin (IgG), as a negative control, and IFNγ-secreting Teff were identified using the IFNγ-catch assay (Fig. 1c and Supplementary Fig. 1c). MAMI- and IgG-reactive IFNγ^+^Teff were isolated as one single cell per well in a plate format using single-cell FACS sorting, and their TCR repertoire was characterized by multiplex RT-PCR, to amplify and sequence the TCRβ chain of each cell separately^39^ without biased enrichment of specific products in both polyclonal and oligoclonal expanded TC populations^40–42^.

MAMI-reactive IFNγ^+^Teff in all tested patients contained a few highly expanded clonotypes (distinct TCRβ nucleotide sequences) with maximum frequencies at 6.8–26.7% (Fig. 1d and Supplementary Fig. 2b). Despite the small number of cells analyzed per patient, the measured proportion of each expanded clonotype among MAMI-IFNγ^+^Teff was higher than the calculated maximum frequency of an undetected clone (*φ*_max_: 0.7–7.2%, Supplementary Table 2). Thus, all relevant extensively expanded MAMI-IFNγ^+^Teff clonotypes were efficiently detected with 95% confidence in all tested patients with the exception of patient 4220 with a lower but still considerably high confidence at 89%. IgG-responding IFNγ^+^Teff also showed oligoclonal expansion of a few prevalent clonotypes displaying frequencies up to 58.3% (Fig. 1e and Supplementary Fig. 2d). *φ*_max_ was estimated at 0.8–3.3% demonstrating a high probability of detecting all major clones expanded at levels above this value (Supplementary Table 2). Nevertheless, MAMI- and IgG-IFNγ^+^Teff shared no or only a few clonotypes (patient 4573: 2, 4578: 19) of intermediate-high prevalence in the IgG-responding subset but of low frequency in the MAMI-reactive population (Supplementary Fig. 3a, b). Consequently, shared clones had a minor impact on the TCR repertoire of the total MAMI-IFNγ^+^Teff subset (4573: 0.1%, 4578: 2.4%). Importantly, the dominant highly expanded MAMI-responding clonotypes were not detected within the IgG-reactive pool, demonstrating that they are independent of unspecific contamination from pre-expanded or activated clones within the tested population.

### Treg and TA-Teff are clonally distinct in patients’ blood

The PB-derived TCR repertoire of Treg from the same patients was assessed on the bulk level using the RNA-based TCR-ligation-anchored-magnetically captured PCR method, which facilitates unbiased ultra-deep high-throughput TCRβ sequencing of populations with both physiological and restricted TCR diversity^24,43,44^. In total, 2942–5443 in-frame Treg TCRβ nucleotide sequences were obtained per patient comprising 214–1645 clonotypes with significant coverage of dominant but also rare clones (*φ*_max_: 0.004–0.02%, Supplementary Table 2). Strikingly, all tested patients contained a few massively expanded Treg clones with maximum frequencies at 2.7–22.9% (Fig. 1f and Supplementary Fig. 2c). To clarify whether dominant Treg clonotypes derive from the observed highly expanded MAMI-reactive IFNγ^+^Teff, we compared the TCRβ repertoire of Treg and MAMI-IFNγ^+^Teff within each patient. Surprisingly, the two subsets shared no clonotypes in four out of five tested patients and only one common clonotype in the fifth patient (4578) (Fig. 1g). The latter was presumably MAMI-specific, as it was not detected among IgG-IFNγ^+^Teff (Supplementary Fig. 3a, c). To quantify the observed overlap, we calculated the Morisita–Horn index (MH), which measures similarity considering not only the number of shared clonotypes between two populations but also their proportion within each population^45^. Considering the low MH value at 0.0003 (Supplementary Table 3), Treg and MAMI-IFNγ^+^Teff are highly distinct. Importantly, the shared clone was of intermediate frequency (4.9%) among MAMI-IFNγ^+^Teff but rare among Treg shaping merely 0.03% of the Treg repertoire, a value below the threshold of sort purity, which was 2.94% in this sample (Fig. 1h and Supplementary Table 1). While patient 4578 had been subjected to neoadjuvant chemotherapy before the operation, all remaining patients suffered from stage I/II breast cancer and had received no previous treatment at the time of the operation (“Methods”, Table 1). Thus, in PB of breast cancer patients, we found no convincing evidence for the conversion of expanded TA-reactive Teff into iTreg at least at early stages of tumor development.

### Oligoclonal expansion of circulating Treg in breast cancer

**Fig. 2:**
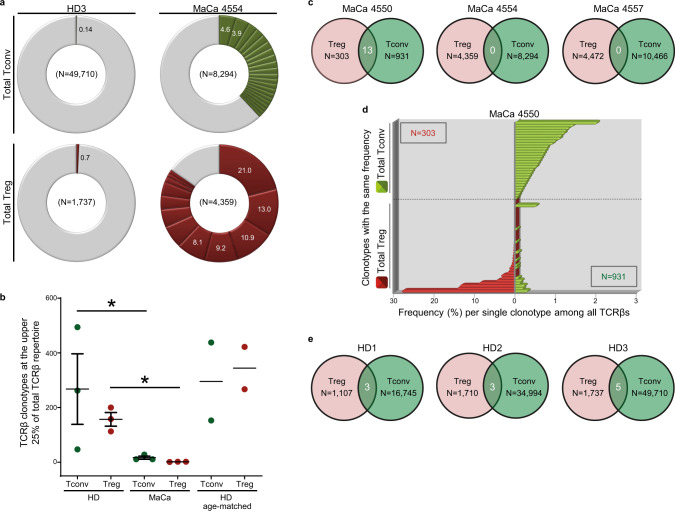
Minor TCR similarity between Tconv and Treg in PB of breast cancer patients and healthy individuals. **a** Pie chart of all clonotypes in order of decreasing percentage (%) among total Tconv (green) and total Treg (red) from one representative breast cancer patient with mammary gland adenocarcinoma (MaCa: *n* = 3) and one healthy donor (HD: *n* = 3), *n* refers to biologically independent replicates. Gray slices correspond to the TCRβ repertoire fraction that contains clonotypes with a frequency below 1%. **b** The absolute number of clonotypes accounting for the highest 25% of the TCRβ repertoire in Treg (red) and Tconv (green) from HD versus MaCa. Cumulative data (HD: *n* = 3, MaCa: *n* = 3, HD age-matched: *n* = 2), *n* refers to biologically independent replicates. Data are presented as mean values for HD age-matched (*n* = 2) or as mean values + /- SEM. *P ≤ 0.05 significant differences between the groups connected with a line, two-sided *t* test on rank data (*P* *=* 0.021 for the comparison of Tconv in three HDs versus three MaCa patients and *P* = 0.021 for the comparison of Treg in three HDs versus three MaCa patients). **c**, **e** Venn diagram of the number of overlapping clonotypes (white) between Treg and Tconv within each (**c**) MaCa patient or (**e**) HD. **d** Frequency of overlapping clonotypes between Treg and Tconv compared to the frequencies of all clonotypes identified per subset in order of decreasing (Treg, red) and increasing (Tconv, green) frequency in patient MaCa 4550. Due to the big number of identified clonotypes per subset, each bar represents a group of clonotypes with identical frequencies (*y* axis) plotted against the frequency of each clonotype in this group (*x* axis). Clonotypes of each subset are separated by a dashed black line. In case of an overlapping clonotype between the two subsets, a red and a green bar align on the *y* axis. *N*, total number of clonotypes among Treg (red) and Tconv (green). Source data are provided as a Source Data file.

To examine whether secondary iTreg stem from CD4^+^Teff that recognize other breast TAs than mammaglobin, we compared the TCR repertoire of total Treg not against a TAA-reactive Teff subset but to the total Tconv population within PB of three additional MaCa patients. To exclude that any observed overlap is due to tumor-irrelevant secondary iTreg generation, the same analysis was performed in three healthy individuals. Using high-throughput bulk TCRβ sequencing^24,43,44^ we obtained 1487–277,467 in-frame TCRβ nucleotide sequences corresponding to 303–49,710 clonotypes per subset with both expanded and scarce clones efficiently detected (*φ*_max_: 0.000007–0.04%, Supplementary Table 2). Tconv and Treg from healthy individuals exhibited a highly diverse TCRβ repertoire with the most prevalent clonotypes detected at 0.14–2.4% within Tconv and 0.6–1.3% among Treg (Fig. 2a and Supplementary Fig. 4a, b). In contrast, tumor patients harbored several extensively expanded Treg clonotypes with maximum frequencies at 12.6–28.1% but also a few slightly expanded Tconv clonotypes at 2–4.6%. Clonotypes expanded above 1% represented the major fraction of the Treg repertoire in tumor patients (4557: 81.2%, 4554: 84.6%, 4550: 85.8%) but only a minor proportion in healthy individuals (HD1: 1.3%, HD2: 0%, HD3: 0%). To exclude that the observed Treg oligoclonality stems from the existing age difference between patients and healthy donors (average 61 and 32, respectively), as TCR diversity decreases with age^46^, we analyzed an additional control group of two older age-matched healthy individuals. Accordingly, Treg TCRβ diversity was overall reduced in tumor patients compared to healthy individuals irrespective of age-matching (Fig. 2b). Thus, patient-derived Treg show a strikingly restricted repertoire dominated by a few prevalent but massively expanded clonotypes, indicating a strong tumor-induced immune-suppressive response that is based on a few TCR specificities in the circulation of breast cancer patients. Interestingly, old age-matched healthy individuals showed increased Treg TCR diversity but similar Tconv TCR diversity compared to young healthy donors. This is in agreement with studies supporting Treg repertoire increase with age through peripheral expansion and conversion from Tconv^47^ but disagrees with the general belief of T-cell repertoire constriction in aging individuals through thymic involution but also antigen selection and clonal expansion in the periphery^48^. This discrepancy may be due to (i) differences in the age “window” of the individuals included in the young or old age group per study, (ii) in the technology used for TCR repertoire characterization but also (iii) in the means used to measure TCR diversity since we assess the number of clones contributing to the upper 25% of the repertoire per group rather than the number of clones identified per specific number of cells or quantity of DNA molecules as performed in other studies^46^. Moreover, previous reports of age-related TCR diversity restriction were based either on the total TC population^46^ or on specific subsets, such as antigen-specific CD8^+^ TC alone^49^ or naive TC^50^, rather than highly pure Treg and Tconv from the CD4^+^ TC compartment alone as in our study. This is further supported by previous reports of age-related clonal restriction mostly affecting the CD8^+^ rather than the CD4^+^ TC subset^48^.

### Circulatory Treg and Tconv are clonally distinct in patients

TCRβ comparison between total Treg and total Tconv within PB of each individual revealed no similarity in two out of three tested patients (Fig. 2c). In the third patient (4550), we detected 13 overlapping clonotypes of high-frequency among Treg but of low-abundance among Tconv (Fig. 2d). The respective MH at 0.023 (Supplementary Table 3) indicated little similarity between Tconv and Treg. Shared clonotypes accounted for merely ~1.78% of the Tconv repertoire, a value only slightly higher than the expected Treg contamination at 1.32% (Supplementary Table 1). Taken together, these data suggest that the observed overlap derives most likely from contaminating highly expanded Treg clones transferred into Tconv during sorting. A similar analysis in PB of healthy individuals revealed only a small number of shared clonotypes between Treg and Tconv (Fig. 2e). The MH was always below 0.001 (Supplementary Table 3), showing that Tconv and Treg are completely dissimilar. Frequencies of overlapping clonotypes were extremely low and below the threshold of sort purity in both Tconv and Treg of each tested healthy individual (HD1: 0.01%, 0.2%; HD2: 0.06%, 0.2%; HD3: 0.01%, 0.4%, respectively, Supplementary Fig. 4b and Supplementary Table 1). Consequently, our study shows no considerable impact of secondary iTreg generation on the Treg repertoire in PB of healthy individuals and breast cancer patients.

### CD4^+^ T-cell single-cell transcriptome sequencing in patients

**Fig. 3:**
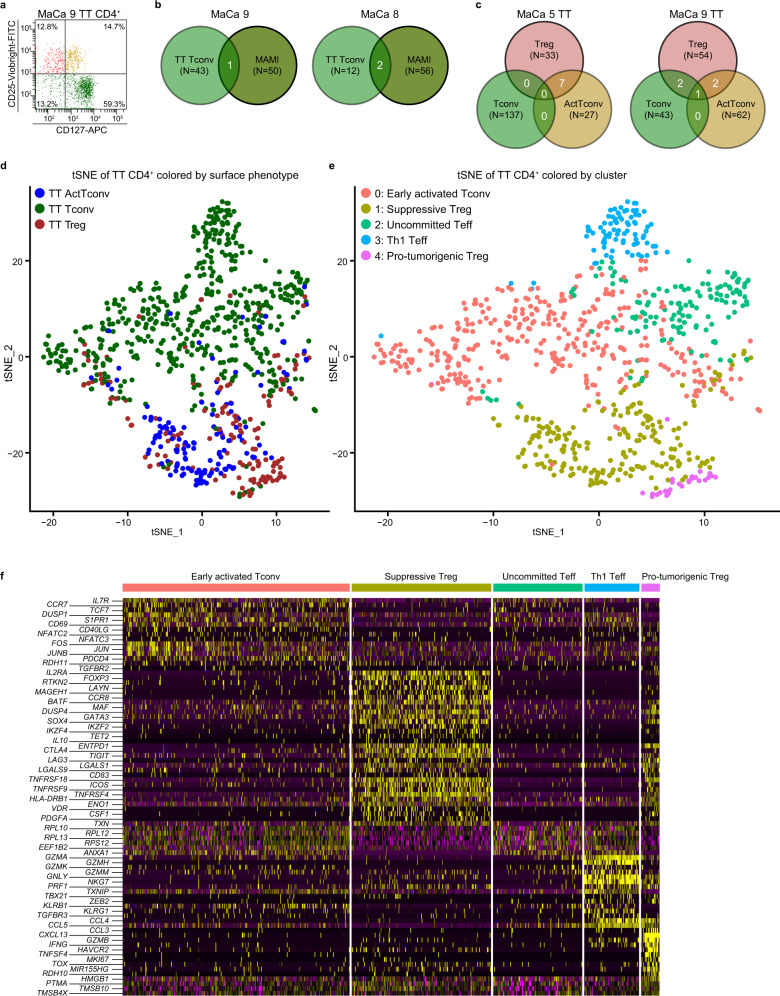
Single-cell transcriptome analysis of circulating TA-reactive and tumor-infiltrating CD4^+^ TC in breast cancer patients. **a** Separation of tumor-infiltrating live CD4^+^ TC into CD25^+^CD127^−^ (Q1, red) Treg, CD25^−^CD127^+/−^ (Q3 and Q4, green) Tconv, and CD25^+^CD127^+^ (Q2, orange) ActTconv using FACS sorting. **b**, **c** Venn diagram showing the number of common (white) TCRαβ clones (unique paired TCRαβ nucleotide sequences) between (**b**) blood-derived MAMI-IFNγ^+^Teff and tumor tissue (TT) infiltrating Tconv or (**c**) between tumor-infiltrating Tconv, Treg and ActTconv per breast cancer patient with mammary gland adenocarcinoma (MaCa). *N*, total number of TCRαβ clones per subset. Representative examples from two out of (**b**) *n* = 3, (**c**) *n* = 4 biologically independent replicates. **d**, **e** t-SNE representation of tumor-infiltrating CD4^+^ TC colored by (**d**) cell-surface phenotype (TT Tconv, dark green; TT Treg, dark red; TT ActTconv, dark blue) or (**e**) transcriptome cluster (0: early activated Tconv, red; 1: suppressive Treg, orange; 2: uncommitted Teff, green; 3: Th1 Teff, blue; 4: Pro-tumorigenic Treg, purple) from *n* = 4 MaCa patients. *n* refers to biologically independent replicates. **f** Heatmap of the top genes differentially expressed (DE) between transcriptome clusters among tumor-infiltrating CD4^+^ TC with high (yellow), intermediate (black), and no expression (purple).

Despite no secondary iTreg detection in patients’ PB, it is possible that antigen-experienced Tconv convert into iTreg within the tumor, where they eventually reside without entering the circulation^18^. To investigate this, we characterized simultaneously the surface phenotype, the TCRαβ sequence and the gene expression profile of CD4^+^ TC from freshly resected breast tumors of four additional MaCa patients using single-cell FACS sorting in combination with single-cell poly(A)-transcriptome sequencing. Tumor-infiltrating TC were stained with fluorescent Abs against the Treg-distinguishing markers CD4, CD25, CD127, and CD45RA^51^ and the CD4^+^ gate was “index sorted” as one cell per well in a plate format. Among tumor-infiltrating CD4^+^ cells, we identified CD25^+^CD127^-^ Treg, CD25^−^CD127^-/+^ Tconv but also a third CD25^+^CD127^+^ subset designated ActTconv (Fig. 3a, Supplementary Figs. 1d and 5a, b), as CD25 is upregulated on TC upon TCR stimulation^52^, and the vast majority lacked CD45RA expression, suggesting previous antigen experience. “Index sorting” records for every single cell its location within the plate along with its size, granularity, and immunofluorescence signals of the Abs stains^53^, allowing retrospective marker expression analysis and confident allocation of every single cell to the Treg or Tconv subset without the need for predefined gates. Thereby, we could isolate all existing intratumoral CD4^+^ cells irrespective of their number (88–352 cells per lesion) or subset frequency and most importantly without background contamination during sorting. To investigate whether iTreg are tumor-reactive, we also performed single-cell sequencing of sorted MAMI-specific Treg and Tconv from autologous blood using either mam_34–48_-labeled HLA Class II tetramers or the IFNγ-secretion assay targeting MAMI-reactive IFNγ^+^Teff (Supplementary Fig. 1e, f and 5c, d). To characterize the gene expression profile of each sorted cell, we performed Smart-Seq2-based single-cell poly(A) RNA amplification and sequencing aiming at ~1.3 million reads per single cell^54,55^. High-quality sequencing data could be obtained for a minimum of 73.2% (patient 8) and a maximum of 92.3% (patient 5) of all single cells sequenced per patient on the basis of retrieved read counts, the number of genes expressed, and the fraction of reads mapping to mitochondrial genes or ERCC spike-ins (Supplementary Fig. 6a). As full-length transcript information is obtained, we applied TraCeR^56^ on the sequencing data to characterize the complete nucleotide sequence of the TCRβ and the TCRα (TRA) chain transcript with a detection efficiency of at least one chain per single cell at 81% and both chains at 56.6% (Supplementary Fig. 7). Circulating Tconv, including MAMI-reactive IFNγ^+^Teff and mam_34–48_-tetramer^+^ Tconv, showed no TCR similarity with Treg from autologous tumors. However, in two out of three tested patients blood-derived MAMI-reactive IFNγ^+^Teff shared identical TCRαβ clones with autologous intratumoral Tconv (Fig. 3b and Supplementary Fig. 8a, e). Interestingly, in patient 8 the most expanded circulating MAMI-reactive IFNγ^+^Teff clone also dominated the tumor-infiltrating Tconv population, indicating a dynamic exchange between the two compartments during a MAMI-specific immune response (Supplementary Fig. 8a). Taken together, our data suggest that peripheral TA-reactive Teff migrate from the blood into the tumor, although the possibility cannot be excluded that intratumoral Tconv egress from the tumor via afferent lymphatic vessels into draining lymph nodes and recirculate in the periphery^57^.

Single-cell transcriptome analysis revealed that tumor-infiltrating CD4^+^ TC and blood-derived MAMI-IFNγ^+^Teff accumulate into two distinct clusters (Supplementary Fig. 6b, c), suggesting that their functional profile is affected by the tumor microenvironment. Within the tumor Treg, Tconv and ActTconv contained several expanded TCRαβ clones (maximum frequency: Treg 6.3%, Tconv 31.3%, ActTconv 7.1%, Supplementary Fig. 7). Identical clones were shared between all three CD4^+^ TC populations (patient 9) but also between Tconv and Treg alone (patient 9) and between ActTconv and Treg (patient 5 and 9), demonstrating that Treg can be converted from ActTconv (Fig. 3c and Supplementary Fig. 8b–e). TCRαβ overlaps were mainly detected among dominant expanded Treg and prevalent ActTconv clones, supporting an association between activation-induced proliferation and phenotype conversion (Supplementary Fig. 8b, c), which most probably reflects the increased probability of abundant clones compared to rare clones to encounter their specific tumor antigen under iTreg-generating conditions, like secreted TGFB, in the local tumor microenvironment.

### Distinct functional clusters among intratumoral CD4^+^ T cells

**Fig. 4:**
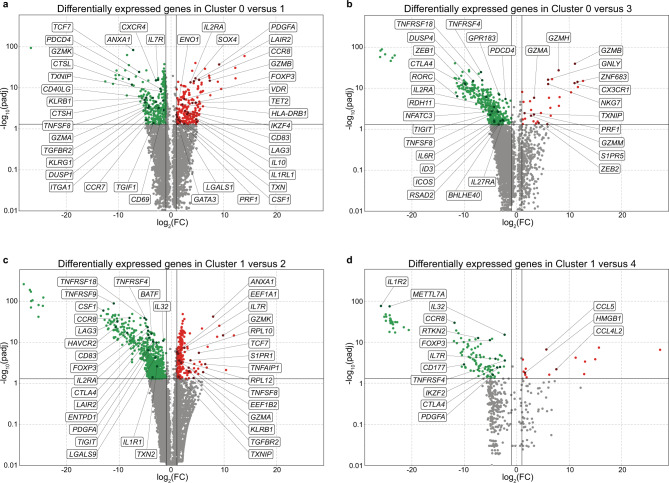
Differentially expressed genes between transcriptome clusters of tumor-infiltrating CD4^+^ TC. **a**–**d** Volcano plots depicting genes differentially expressed between transcriptome clusters of tumor-infiltrating CD4^+^ TC from *n* = 4 breast cancer patients. *n*, biologically independent replicates. Pairwise comparison between (**a**) cluster 0 (green) and cluster 1 (red), (**b**) cluster 0 (green) and cluster 3 (red), (**c**) cluster 1 (green) and cluster 2 (red), and (**d**) cluster 1 (green) and cluster 4 (red) using two-class differential expression analysis with the function DESeq with default parameters, which performs negative-binomial generalized linear model fitting using Wald tests for significance. FC fold change, padj adjusted *P* values.

On the basis of their gene expression profile tumor-infiltrating CD4^+^ TC accumulated into five well-separated clusters (Fig. 3d, e) defined by the expression of 1662 significant marker genes (*P* < 0.05, cluster 0:154, 1:546, 2:58, 3:114, 4:790, Supplementary Fig. 6d). CD25^−^CD127^−/+^ Tconv were grouped into clusters 0, 2, and 3, CD25^+^CD127^+^ ActTconv were detected in cluster 1, while CD25^+^CD127^−^ Treg were predominantly segregated in clusters 4 and 1. In accordance, clusters 0, 2, and 3 were marked by enhanced *IL7R* but reduced *FOXP3* and *IL2RA* transcription, confirming their Tconv phenotype (Fig. 3f and Supplementary Fig. 9e). Particularly, cluster 0 showed increased expression of *CCR7*, disposing of a central memory profile^58^, but also *ID3* and *ZEB1*, which are crucial for memory TC differentiation survival and function^59,60^ together with the stemness controlling factor *TCF-7*^[ 61^, rather supporting a self-renewing memory phenotype (Figs. 3f and 4a, b, Supplementary Figs. 6d, 9a, b, e and 10). Among its dominant markers was *DUSP1*, which is essential for functional Th1 and Th17 CD4^+^ TC differentiation but also the production of the Th1 cytokines IFNγ and TNFα^62^. Apart from Th1 promoting genes like *IL27RA*^63^ and *BHLHE40*, which is indispensable for optimal IFNγ production irrespective of the induction of the master Th1 transcription factor *T-bet* (*TBX21*)^64^, cluster 0 upregulated *GPR183* facilitating Tfh development^65^, *RORC* and *IL6R* supporting Th17 differentiation^66^, *RSAD2* associated with Th2 generation^67^ and *FOXP1*, which sustains naïve TC quiescence^68^ and inhibits Tfh differentiation^69^. Importantly, *TGFBR2*, the receptor for TGFB, and *RDH11*, an enzyme mediating the production of Retinoic Acid, were also enhanced in cluster 0, suggesting its potential for iTreg conversion^14^. In addition, cluster 0 expressed several tissue-resident markers, including *ITGA1*, *S1PR1,* and *CD69*^70^, which is also an early activation TC marker^71^. The activated state of cluster 0 was further confirmed by enhanced levels of *CD40L*, *TNFSF8* (*CD30L*)^72^, *ICOS*, *NFATC2*, *NFATC3* but also *FOSB*, *JUN,* and *JUNB*, subunits of the AP-1 transcription factor, which is undetectable in unstimulated TC but rapidly induced upon activation. Interestingly, *PDCD4*, which is known to suppress AP-1 transactivation, induce apoptosis, and inhibit protein synthesis, was also highly transcribed^73^. Taken together, cluster 0 contained predominantly central memory tissue-resident Tconv at the early stages of activation and at the verge of differentiation between Th1, Th2, Th17, Tfh, or quiescence but also towards iTreg. Thus, cluster 0 was designated early activated Tconv cluster 0.

Cluster 2 was characterized by 30 ribosomal proteins, e.g., *RPL12*, known to be massively transcribed on CD4^+^Teff following TCR stimulation to facilitate increased cytokine production^74^, but also *ANXA1* which dampens proliferation and Th1/Th17 cytokine release mediating attenuation of TC-driven inflammatory responses^75^ (Figs. 3f and 4c, Supplementary Figs. 6d, 9a and 10). Interestingly, we did not detect the marked expression of effector genes associated with common TC effector lineages. Furthermore, it upregulated *TGFBR2*, increasing the possibility of iTreg conversion within the tumor. Thus, cluster 2 is referred to as uncommitted Teff cluster 2.

Cluster 3 was characterized by *S1PR5*, which promotes egress from the lymph nodes and the bone marrow^76^ and the tissue-retention markers *ZNF683* and *CX3CR1*, which facilitate migration to tumors and inflamed endothelium^77^ (Fig. 4b, Supplementary Fig. 6d, 9c, d and 10). The increased expression of cytotoxic effector molecules *GZMA*, *GZMB*, *GZMH*, *GZMM*, *GNLY*, *NKG7*, and *PRF1*^78^ but also of the thioredoxin function inhibitor *TXNIP*^79^, demonstrates a potent effector capacity to eliminate malignant cells and suppress tumor growth (Fig. 3f and Supplementary Fig. 9e). Furthermore, cluster 3 expressed *TBX21* and *ZEB2*, which co-operate to drive cytotoxic Teff differentiation, *IFNG*, *KLRB1,* and *KLRG1* indicative of terminally differentiated Teff with increased killing capacity and IFNγ secretion but decreased proliferation, longevity, and plasticity^80^. Therefore, cluster 3 was characterized as Th1 cluster 3. Interestingly, *TGFBR3*, co-receptor for TGFB involved in iTreg generation^81^, was a significant marker gene of cluster 3, supporting the increased potential for conversion into suppressive cells upon TGFB production in the tumor.

Cluster 1 (phenotyped as ActTconv and Treg) was defined by increased transcription of the typical Treg markers *IL2RA* and *FOXP3* and in many cells simultaneous expression of *IL7R*, however at levels significantly lower than Tconv cluster 0, 2 or 3. (Figs. 3f and 4a, c, d, Supplementary Figs. 6d, 9c, e and 10). Importantly, cluster 1 was also marked by *RTKN2*, which was recently identified in healthy donor blood-derived Treg^82^, and the tumor-infiltrating Treg markers *LAYN*, *MAGEH1*, and *CCR8*, which associates with increased proliferative potential and suppressive capacity but most importantly poor prognosis in breast cancer, NSCLC, and CRC patients^30,83^. Furthermore, cluster 1 exhibited increased expression of *IL1R2* and the IL-33 receptor ST2 (*IL1RL1*), which enhances the proportion and suppressive potential of tumor-infiltrating Treg in HNSCC patients^84^. Cluster 1 was enriched with the inflammation-associated genes *IL-32* and transcription factor *BATF*, several tissue-resident Treg markers like *MAF*^85^, the Treg-associated transcription factors *GATA3*, *DUSP4*, *IKZF4* (*Eos*), *SOX4*, *IKZF2* (*Helios*) and *TET2*, which regulates TSDR *FOXP3* demethylation inhibiting conversion to Th17 TC^86^. Furthermore, this cluster exhibited a plethora of suppressive effector Treg molecules including *GZMB*, *PRF1*, *IL10*, *ENTPD1* (*CD39*), *CTLA4*, *TIGIT*, *LAG3*, *LGALS1*, *LGAL9*, and *CD83*, markers of enhanced activation like *ICOS*, *TNFRSF18* (*GITR*), *TNFRSF4* (*OX40*), *TNFRSF9* (*4-1BB*), and *HLA-DRs*^3,87,88^ but also the soluble receptor for collagen *LAIR2*. Cluster 1 also upregulated genes reported to facilitate the conversion of human Tconv into iTreg such as *ENO1*^89^ and *VDR*^90^. Finally, *PDGFA* and *CSF-1* were highly expressed in this cluster suggesting tissue-remodeling properties essential for wound healing^91,92^, which combined with upregulated *TXN*^93^ improve their capacity to create an immunotolerant environment and promote tumor progression. Taken together, this cluster contains CD4^+^ cells of an activated suppressive phenotype resembling Treg, despite the co-expression of CD127 by a proportion of cells in this cluster.

Cluster 4 was defined by increased *IL2RA* transcription but significant *IL7R* downregulation even compared to suppressive cluster 1, confirming its CD25^+^CD127^−^ Treg surface phenotype (Figs. 3f and 4d, Supplementary Fig. 6d, 9b, d, e and 10). Similar to cluster 1, cluster 4 exhibited profound upregulation of typical Treg effector molecules like *CD39*, *HAVCR2* (*TIM3*), *TIGIT*, *LAG3*, *CD83*, *LGAL1*, *LGAL9*, *GZMB*, and *PRF1*, demonstrating a potent immune-suppressive phenotype. Increased levels of *GITR*, *TNFSF4* (*OX40L*), and *4-1BB* suggest activation, while increased *MKI67* expression identifies cluster 4 as the most proliferative cluster in the tumor. Despite the expression of the exhaustion marker *TOX*^94^, the genes *IFNG*, *ZEB2*, *BHLHE40*, and *TBX21* were also highly transcribed, suggesting a Th1-like effector Treg suppressive phenotype. Although cluster 4 contained only a minority of *FOXP3*-transcribing cells, among its markers were *RUNX3*, which binds to the *FOXP3* promoter inducing its transcription^95^, *DUSP4* which stabilizes FOXP3 expression^96^ and *MIR155HG*, which is induced by FOXP3 and associates with breast tumor initiation^97^. Taken together, these data suggest that despite low transcript levels, this cluster can express FOXP3. Furthermore, it exhibited enhanced levels of *RDH10*, an enzyme essential for the production of Retinoic Acid, which suppresses Th1 and Th17 differentiation but promotes the conversion of Tconv into iTreg^98^. Among its significant markers were *CCL5*, *CCL3*, and *CCL4*, which chemoattract CD8^+^ TC and Treg^99^, and the B-cell chemotactic molecule *CXCL13*, which facilitates B-cell homing to follicles. However, the lack of *CXCR5* and *Bcl6* expression points against its designation as classic Tfh, while the upregulation of *SOX4* and *PDCD1* supports their contribution in tertiary lymphoid-like structure generation under inflammation^100^. Compared to cluster 1, cluster 4 showed increased expression of *HMGB1*, which dampens IFNγ release by Tconv but is also a chemoattractant, functional enhancer, and inducer of Treg^101,102^. Additionally, it upregulated *PTMA* and *TMSB10*, which correlate with poor prognosis in breast cancer^103,104^ and *TMSB4X*, which promotes tumorigenesis through TGFB^105^. Thus, cluster 4 contains immune-suppressive Treg with pronounced tumor-promoting properties. As cluster 4 analysis was based on a limited number of cells, further studies are necessary to confirm our findings and understand deeper their function in the tumor microenvironment.

### Early activated Tconv convert to Th1 Teff or iTreg in tumors

**Fig. 5:**
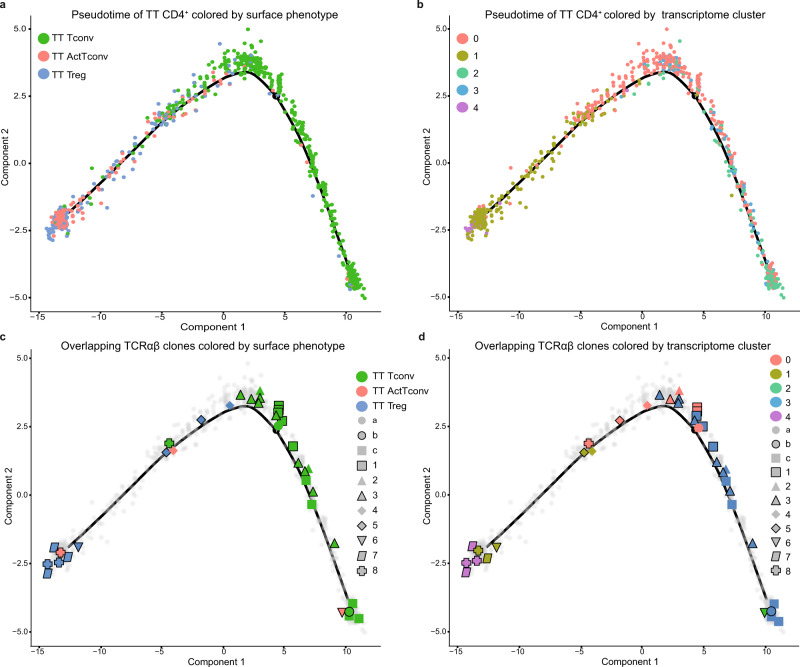
Monocle single-cell trajectory of tumor-infiltrating CD4^+^ TC from breast cancer patients. Pseudotime transcriptome analysis of tumor tissue (TT)-derived CD4^+^ TC from four patients. **a**, **b** All TT CD4^+^ TC are depicted colored by (**a**) cell-surface phenotype (TT Tconv, green; TT ActTconv, red; TT Treg, blue) or (**b**) single-cell transcriptome cluster (0: red, 1: orange, 2: green, 3: blue, 4: purple). **c**, **d** All expanded (n≥2 single cells) TCRαβ clones (unique paired TCRαβ nucleotide sequences) are depicted colored by (**c**) cell-surface phenotype (TT Tconv, green; TT ActTconv, red; TT Treg, blue) or (**d**) transcriptome cluster (0: red, 1: orange, 2: green, 3: blue, 4: purple) on a gray background of unexpanded clones (*n* = 1 single-cell). Each particular shape corresponds to a different unique TCRαβ clone. TCRαβ clones a-c are shared between tumor-infiltrating Tconv and circulating MAMI-reactive IFNγ^+^Teff, while clones 1–8 are shared between different (**c**) cell-surface phenotypes or (**d**) clusters within the tumor.

To understand the phenotypic transitions between the five transcriptional clusters of tumor-infiltrating CD4^+^ TC, we performed pseudotime ordering, which revealed that early activated Tconv cluster 0 split into three branches representing three distinct developmental states (Fig. 5a, b and Supplementary Fig. 11a). The central branch-state 3 included mostly early activated Tconv cluster 0, branch-state 2 contained Teff cluster 2 and Th1 cluster 3, while branch-state 1 contained cluster 0 followed by both suppressive Treg clusters 1 and 4. Taken together, monocle analysis suggests that upon activation in the tumor tissue Tconv differentiate either towards tumor-eliminating effector Th1 cells or towards tumor-promoting suppressive Treg. To ascertain the validity of this observation, we also performed pseudotime analysis using Slingshot. As shown in Supplementary Fig. 11b–d, Slingshot confirms that early activated Tconv cluster 0 comprises the central point of the trajectory developing, on the one side, toward uncommitted and Th1 Teff and, on the other side, toward suppressive and pro-tumorigenic Treg.

Interestingly, the blood-derived MAMI-reactive IFNγ^+^Teff TCRαβ clones (a, b, c), which overlap with tumor-infiltrating Tconv (Fig. 3b and Supplementary Fig. 8a), were detected in the early activated Tconv cluster 0 or the Th1 cluster 3 (Fig. 5c, d and Supplementary Fig. 12). However, within the tumor identical TCRαβ clones were shared between the early activation cluster 0 and the terminally differentiated Th1 cluster 3 (TCRαβ 1, 2 and 3) across the first trajectory branch (Fig. 5d), showing that TA-reactive Tconv upon their entry into the tumor can become activated and develop into potent tumor-killing Teff. At the same time, across the second trajectory branch, we detected common clones between the early activation Tconv cluster 0 and the activated suppressive Treg cluster 1 (TCRαβ 4, 5) but also between Tconv cluster 0, activated suppressive Treg cluster 1, and suppressive tumor-promoting Treg cluster 4 (TCRαβ 8), while one clone (TCRαβ 7) was shared between clusters 1 and 4 (Fig. 5d and Supplementary Fig. 12), supporting that upon activation within the tumor Tconv can differentiate into potent immune suppressors. Importantly, the uncommitted Teff cluster 2 on the first branch and the activated suppressive Treg cluster 1 on the second branch contained one identical clone (TCRαβ 6), further supporting that intratumoral Teff and suppressive Treg can be the progeny of the same expanded clone (Fig. 5d and Supplementary Fig. 12). This conclusion was enhanced by additional overlaps between cells that shared only one of the TCRαβ chains, as the second remained undetected (Supplementary Fig. 12). Consequently, our data suggest clonal interconversion between Tconv and suppressive tumor-promoting Treg through an intermediate activated state.

### Major TCR overlap between Treg and Tconv in breast tumors

**Fig. 6:**
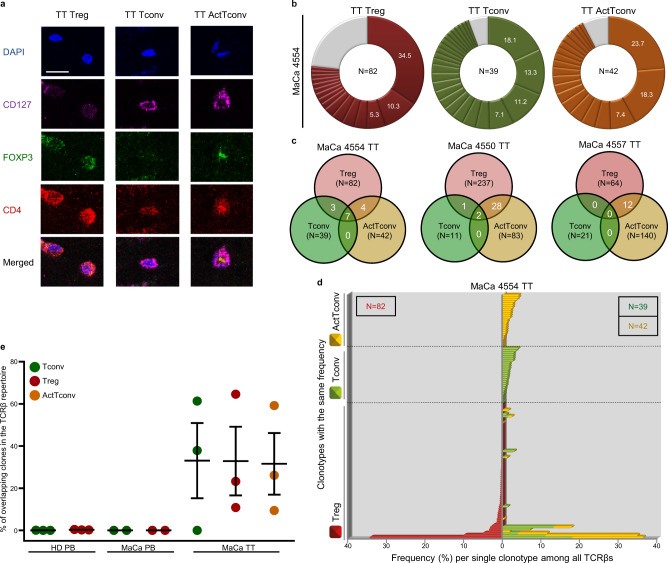
Major overlap between Treg and Tconv subsets in breast tumors. **a** Representative immunostaining of FFPE breast tumor tissue (TT) from *n* = 3 biologically independent replicates. CD4 (red), CD127 (purple), FOXP3 (green), DAPI (blue). TT Treg (CD4^+^FOXP3^+^CD127^−^), TT Tconv (CD4^+^FOXP3^-^CD127^+^), TT ActTconv (CD4^+^FOXP3^+^CD127^+^). Scale bar denotes 2.32 µm. **b** Pie chart of all clonotypes (unique TCRβ nucleotide sequences) detected among TT Treg (red), TT Tconv (green), and TT ActTconv (orange) in order of decreasing percentage (%). Gray slices correspond to the TCRβ repertoire fraction that contains clonotypes with a frequency below 1%. Representative data from breast cancer patient with mammary gland adenocarcinoma MaCa 4554 from *n* = 3 biologically independent replicates. **c** Venn diagram of shared clonotypes (white) between TT Treg, TT Tconv, and TT ActTconv within each patient. **d** Frequency of common clonotypes between TT Treg, TT Tconv, and TT ActTconv in relation to the frequency distribution of all clonotypes per subset. *N*, total number of clonotypes recovered from TT Treg (red), TT Tconv (green), and TT ActTconv (orange). **e** Total frequency of Treg clonotypes overlapping with Tconv among Tconv (green), with Tconv and ActTconv among Treg (red) or with ActTconv among ActTconv (orange) either in peripheral blood (PB) or within TT in *n* = 3 MaCa patients and *n* = 3 healthy individuals (HD) (cumulative data, mean ± s.e.m.). *n* refers to biologically independent replicates. MaCa 4550 data obtained from Treg and Tconv in PB were excluded, as the observed overlap between the two subsets could be explained by background contamination during sorting, as explained in the main text of the manuscript. Source data are provided as a Source Data file.

While deep single-cell poly(A)-transcriptome sequencing allowed for accurate functional characterization of circulating and intratumoral CD4^+^ populations, the relatively low number of analyzed cells per tumor entails the risk of underestimating clonal conversion between distinct CD4^+^ subsets in local niches of the tumor microenvironment due to variations in inflammation and hypoxia^106,107^. We, therefore, sought to analyze intratumoral secondary iTreg generation from Tconv in connection with their spatial distribution within the lesion. To this end, we obtained FFPE tumor tissue from the same breast cancer patients assessed before for TCR clonality in PB (Fig. 2c) and performed Immunofluorescence analysis on serial tissue sections against CD4, FOXP3, and CD127 (Fig. 6a). Of note, all three tumor samples were obtained upon resection from early-stage I/II breast cancer patients without any neoadjuvant pre-treatment (“Methods”, Table 1). Tumor-infiltrating CD4^+^ cells consisted of FOXP3^+^CD127^−^ Treg, FOXP3^−^CD127^+^ Tconv but also a major proportion of FOXP3^+^CD127^+^ ActTconv, which according to the single-cell transcriptome analysis above resemble cluster 1 and, thus, possess an activated phenotype with immune-suppressive properties. Using highly precise single-cell laser microdissection, we could isolate neighboring Treg, Tconv, and ActTconv from local tumor niches at ~1000 cells per subset followed by gDNA-based TCRβ high-throughput sequencing^108^ (Supplementary Table 4), to characterize the TCR repertoire of each subset separately. Single-cell transcriptome data revealed no considerable difference in similarity (MH index) between tumor-infiltrating subsets on the basis of the TCRβ sequence alone compared to paired TCRαβ sequences per single cell (Supplementary Fig. 8e), demonstrating that TCRβ diversity and overlap largely reflects the true TCRαβ repertoire in tumor lesions. Compared to the highly diverse TCR repertoire of total intratumoral TC, all three subsets exhibited a few massively expanded clonotypes (maximal frequency Treg: 34.5%, Tconv: 24.1%, ActTconv: 58.8%, Fig. 6b and Supplementary Fig. 13a–c). Interestingly, in two out of three patients we found TCRβ nucleotide sequences shared by all three subsets (Fig. 6c), supporting a yet undefined tumor-driven local interconversion mechanism between Tconv, ActTconv, and Treg.

Shared sequences between the subsets were restricted to highly expanded Tconv clones which overlapped with both prevalent and rare Treg clonotypes (Fig. 6d and Supplementary Fig. 13d), suggesting that dominant antigen-experienced Tconv clones can differentiate to variable extents into secondary iTreg. Likewise, dominant Tconv were identical with high- but also low-frequency ActTconv clonotypes, and major ActTconv clones were overlapping with prominent and scarce Treg clonotypes (Fig. 6d and Supplementary Fig. 13d, e), implying that expanded Tconv convert into activated suppressive TC and these, respectively, into Treg also to variable extents. Interestingly, in two out of three patients a large proportion of the Tconv subset (patient 4554: 61.3%, 4550: 37.9%, Fig. 6d, e and Supplementary Fig. 13d) follows the 2^nd^ arm of the monocle trajectory towards suppressive and tumor-promoting Treg, while all Tconv of patient 4557 (Supplementary Fig. 13e) appear to follow the 1^st^ arm of the monocle trajectory towards Th1 Teff development, as we did not observe overlaps between Tconv and Treg in this tumor. No patient showed common clonotypes between Tconv and Treg alone without additional overlap with activated suppressive TC from cluster 1. Notwithstanding, shared clones between Treg and activated suppressive TC were not detected among Tconv in patient 4557 (Fig. 6c and Supplementary Fig. 13e), suggesting that the acquisition of a FOXP3^+^CD127^+^ activated phenotype is required for Tconv conversion into secondary iTreg. Taken together, these observations suggest that secondary iTreg-infiltrates in tumors are generated from ActTconv. Overlapping clonotypes represented a large proportion (10.8–64.7%) of intratumoral Treg but were not identified among circulating Treg of the same patient (Fig. 6e, Supplementary Fig. 13f, and Supplementary Table 3), showing that secondary iTreg generated within the tumor rarely enter the circulation. Interestingly, tumor and blood had completely distinct Treg repertoires (Supplementary Table 3), suggesting that intratumoral Treg do not derive from circulating Treg during early-stage I/II tumor development in breast cancer patients. Despite no clonal overlap between Treg in the tumor and Tconv in peripheral blood (Supplementary Table 3), circulating Tconv were detected within the tumor (Supplementary Fig. 13g, h and Supplementary Table 3), suggesting that intratumoral secondary iTreg are converted from immigrated circulatory Tconv.

### Treg functional stability in human breast tumors

While Treg are considered a terminally differentiated and functionally stable lineage, several studies suggest that under lymphopenic or inflammatory conditions uncommitted Treg lose both FOXP3 expression and their suppressive identity and acquire pathogenic effector activity^109^. Treg lineage commitment and functional maintenance are regulated by the CpG methylation status of the conserved noncoding sequence CNS2 inside the *FOXP3* locus^13^. CNS2 is demethylated in nTreg, allowing stable FOXP3 expression and suppressive function, but completely methylated in Tconv even during transient FOXP3 expression upon activation^110^. Interestingly, iTreg generated in vitro by TGFB show only partial CNS2 demethylation and functional plasticity^111^. However, in vivo generated iTreg bear almost completely demethylated CNS2 and retain their suppressive character^112^. While CNS2 methylation of FOXP3^+^ suppressive iTreg in breast tumors can efficiently indicate their functional stability and, consequently, their contribution to immune-suppression within the tumor, the small size of obtained clinical samples and technical limitations of DNA methylation analysis at the single-cell level render this approach extremely challenging. Nevertheless, the depth of single-cell transcriptome analysis performed in our study revealed that *FOXP3*^+^ iTreg (suppressive Treg cluster 1) express not only a plethora of genes with well-established immune-suppressive function and tumor-promoting activity but also genes that support a committed FOXP3^+^ iTreg phenotype. Among these genes is *TET2* (Fig. 4a and Supplementary Fig. 10g), whose expression in Treg inhibits CNS2 hypermethylation, conversion to Th17 effectors during severe inflammation but also upregulation of genes associated with cell cycle, DNA damage, and cancer^113^. Furthermore, *FOXP3*^+^ iTreg in our study were characterized by the marker gene *PRDM1* (*BLIMP1*) (Supplementary Fig. 10g), which prevents *FOXP3* hypermethylation under inflammatory conditions^114^ and *TNFRSF1B* (Supplementary Fig. 10g), commonly known as *TNFR2*, which prevents not only *FOXP3* promoter methylation but also Treg conversion into pathogenic effectors^115^. Thus, FOXP3^+^ suppressive iTreg sharing TCR clones with Tconv in breast tumors possess protective machinery against FOXP3 methylation supporting their functional stability.

## Discussion

In summary, we show no clonal relationship between circulatory and tumor-resident Treg in breast cancer patients, although both subsets are characterized by the massive expansion of a few dominant clones. In contrast, circulating expanded Tconv clones, including TA-reactive Th1 cells, are also prevalent in the tumor tissue, suggesting their capacity to infiltrate the tumor. Single-cell transcriptome analysis revealed the presence of five functionally distinct CD4^+^ TC clusters within the tumor, comprising early activated Tconv, uncommitted Teff, Th1 Teff but also immune-suppressive and tumor-promoting Treg, while other helper TC lineages were negligible. Tumor-infiltrating Tconv, especially the early activated subset, showed expansion and marked clonal overlap with cells of a Th1 Teff phenotype but also with dominant clones of a suppressive bona fide Treg phenotype, demonstrating common clonal origin between classical Tconv and suppressive Treg in human breast tumors. Pseudotime analysis suggests the presence of two major differentiation trajectories within the tumor, both originating from early activated Tconv, developing either into Th1 Teff or into pro-tumorigenic suppressive Treg in association with genes required for Tconv conversion into iTreg expressed along this trajectory. Single-cell laser microdissection analysis shows that only one or both trajectories may be active within each tumor depending on the microenvironment. Taken together, our data suggest that secondary iTreg generation occurs after TCR-dependent activation of few highly expanded Tconv within the microenvironment of breast tumors and can considerably contribute to the repertoire of tumor-infiltrating Treg. Further studies to confirm this process and to elucidate the factors dictating tumor-specific iTreg generation will improve our understanding of Treg accumulation and functions in cancer patients and, consequently, the design of efficient cancer immunotherapies in the future.

## Methods

### Healthy donors and breast cancer patients

Peripheral blood (PB) of healthy donors (HD) was provided by the Blood Donation Centre of the University of Heidelberg. PB from breast cancer patients with mammary gland adenocarcinoma (MaCa) was obtained upon primary tumor resection at the Gynecological Hospital of the University of Heidelberg. Primary breast tumors from the same patients were retrieved either fresh upon resection or as formalin-fixed paraffin-embedded (FFPE) tumor tissue (TT) from the Gynecological Hospital of the University of Heidelberg. Tumor tissue from tested patients showed low-intermediate binding of the specific anti-mammaglobin antibody clone 31A5 (Ventana, 0.05 µg ml^−1^) by Immunohistochemistry, which was performed by the Pathology Department in the University Medical Centre of Heidelberg. PB and tissue samples were used after informed consent of all included individuals and the protocol was approved by the Ethics Committee of the University Medical Centre of Heidelberg (-L-225/2003, -S-293-2011). *HLA-DRB1* locus typing of patients was performed using PCR by the Department of Immunology of the University of Heidelberg.

**Table 1:** TNM stage, previous treatment, and tissue analyzed from tested breast cancer patients upon tumor resection.

Tested individual	Tumor tissue	Peripheral blood	TNM	Neoadjuvant chemotherapy
MaCa 4550	Yes	Yes	pT1c (m), pN0 (0/4 sn), L0 G2 R0	No
MaCa 4554	Yes	Yes	pT2 (m), pN1a (1/17), R0	No
MaCa 4557	Yes	Yes	pT1c, pN0 (0/1 sn), L0, R0	No
MaCa 4220	No	Yes	pT1c (m), pN0 (0/2 sn), L0, R0	No
MaCa 4223	No	Yes	pT2, pN2a (7/17), L1, R0	No
MaCa 4572	No	Yes	pT2 pN0(0/17) R0 (2005)	No
MaCa 4573	No	Yes	(right) pT1c pN0 (0/8) L0 R0 (left) pT1a, pN0 (0/2 sn), L0, R0	No
MaCa 4578	No	Yes	cT4d, cN0,ypT0, ypN0 (0/22), L0, R0	Yes
MaCa 5	Yes	Yes	cT4b cN+ M1 (HEP), ypT3(2) ypN1a(2/23) pM1 (HEP) L0 R0	Yes
MaCa 6	No	Yes	cT4b cN+ M1 (HEP, PUL)	No
MaCa 7	Yes	Yes	cT4d, cN+ , ypT1mic, ypN0(0/14), L0, R0	Yes
MaCa 8	Yes	Yes	(left) cT4(m) cN+ , ypT0, ypN0(0/19), L0, R0	Yes
MaCa 9	Yes	Yes	pT3, pN0 (0/4 sn), L0, G3, RX	No

*MaCa,* breast cancer patient.

### Peptides

Synthetic 50 amino acid-long polypeptides served as test antigens. MAMI p4–56: LMVLMLAALSQHCYAGSGCPLLENVISKTINPQVSKTEYKELLQEFIDDNATT derived from mammaglobin was used as breast TAA. Human immunoglobulin IgG p40–89: SWNSGALTSGVHTFPAVLQSSGLYSLSSVVTVPSSSLGTQTYICNVNHKP acted as a negative control antigen.

### T-cell/dendritic cell isolation and culture from PB

PB mononuclear cells (PBMC) were isolated using Ficoll gradient centrifugation from the white interphase between Biocoll separating solution (Biochrom) and plasma. PBMC were incubated in X-VIVO 20 medium (BioWhittaker, LONZA) on Tissue Culture Petri dishes (TPP) for 1–2 h to separate between adherent monocytes and T cells (TC) in suspension. Nonadherent cells were collected and cultured further in RPMI medium (PAA) with 10% AB serum (PAA Laboratories), 1% HEPES (PAN-Biotech), 100 U ml^−1^ rIL-2 (Chiron) and 60 U ml^−1^ rIL-4 (PromoCell) for 5 to 7 days. One day before antigen-specific TC activation with peptide-loaded dendritic cells (DC), TC-enriched nonadherent cells were transferred into X-VIVO 20 medium (BioWhittaker, LONZA) alone without any additional cytokines. Consequently, TC were depleted from contaminating cells using the Dynal T cell Negative Isolation Kit or the Dynabeads Untouched Human T Cell Kit (11344D, Dynal, Invitrogen, 1:6).

PBMC-derived adherent cells were used to differentiate monocytes into mature DC after culture in X-VIVO 20 supplemented with 2% autologous serum, 560 U ml^−1^ GM-CSF (Berlex, Bayer-Schering Pharma), and 1000 U ml^−1^ rIL-4 (PromoCell) for 5 to 7 days until the generation of visible dendrites^116^. Mature DC were purified from contaminating B cells, TC and NK cells using Dynabeads Pan Mouse IgG (11042, Dynal, Invitrogen) combined with mouse IgG anti-human Abs against CD19 (clone HD37, Dr. G. Moldenhauer, Heidelberg, 1:26), CD3 (clone OKT3, Dr. G. Moldenhauer, Heidelberg, 1:26) and CD56 (clone C218, Beckman Coulter, 1:6)^117^.

### Isolation of conventional and regulatory TC from PB using FACS Sorting

Purified TC from PB were firstly treated with human Ig (Sandoglobulin, CSL Behring AG, PZN-0571760, 1.5 mg ml^−1^) in FACS buffer, namely PBS (PAA Laboratories) supplemented with 2% FCS (Biochrom), to block Fc-receptors, as possible sites of unspecific Ab binding. Cell-surface marker staining was performed in MACS buffer—PBS including 0.5% AB serum (PAA Laboratories) and 2 mM EDTA (Biochrom)—in two sequential steps. First, TC were incubated with anti-CD25-biotin (clone 4E3, Miltenyi Biotec, 1:10) and anti-CD127-APC (clone MB15-18C9, Miltenyi Biotec, 1:10) for 10 min at 4–8 °C. After washing with MACS buffer, TC were further stained with anti-biotin-PerCP (clone Bio3-18E7, Miltenyi Biotec, 1:10) and anti-CD4-APC-Cy7 (clone RPA-T4, BD Biosciences, 1:20) for 10 min at 4–8 °C. Consequently, the cells were washed, single-cell filtered, and resuspended in FACS buffer supplemented with Propidium Iodide (PI, BD Biosciences, 1:20) for dead-cell exclusion. Finally, live CD4^+^ cells were separated into conventional TC (Tconv, CD4^+^CD25^−^CD127^+/−^) and regulatory TC (Treg, CD4^+^CD25^+^CD127^−/low^) using a Flow Cytometer and Cell Sorter FACSAria I, II, or III (BD Biosciences) with FACSDiva software (version 6.1.3 and 8.0.1, BD Biosciences). To measure contamination of Treg in sorted Tconv or of Tconv in sorted Treg, both sorted populations were re-analyzed using the same sorter, gates, and settings, as during sorting.

### Intracellular FOXP3 staining

To test the purity of live sorted Tconv and Treg populations on the basis of FOXP3 expression, we performed intracellular FOXP3 staining directly after sorting. The sorted subsets were first washed with PBS (PAA laboratories) and treated with the LIVE⁄DEAD Fixable Dead Cell Yellow Stain Kit (cat. L-34959, Invitrogen) for dead-cell exclusion among fixed cells. Subsequently, the cells were fixed and permeabilized using a mixture of the FOXP3 Fixation/Permeabilization Concentrate and Diluent (cat. 00-5521, eBioscience) at a ratio of 1:3 followed by treatment with the Permeabilization buffer (cat. 00-8333, eBioscience). Finally, fixed cells were stained with anti-FOXP3-PE/eFluor 450 (clone 236A/E7, eBioscience, 1:10) according to the manufacturer’s recommendations. Fluorescently labeled populations were analyzed in a Flow Cytometer FACSCanto II (BD Biosciences) with FACSDiva software (Version 6.1.3, BD Biosciences).

### Total RNA purification

The purification of total RNA from sorted Tconv or Treg in PB of breast cancer patients or healthy individuals was performed directly after sorting using either the RNeasy Mini Kit (Qiagen) or the RNeasy Micro Kit (Qiagen) for cell numbers lower than 5 × 10^5^.

### Isolation of IFNγ^+^ effector CD4^+^ TC using the IFNγ secretion assay and single-cell FACS sorting

Tumor-antigen-reactive IFNγ-producing effector CD4^+^ TC (Teff) were identified using the IFNγ Secretion Assay- Detection Kit (PE) human (cat. 130-054-202, Miltenyi Biotec) according to the manufacturer’s recommendations with slight modifications. In particular, purified DC from PB of breast cancer patients were transferred into 96-well plates, cultured in X-VIVO 20 (BioWhittaker, LONZA) alone, and pulsed with 20 µg of MAMI or IgG polypeptide per 2 × 10^4^ DC at 37 °C for ~12 h. MAMI or IgG-pulsed DC were then co-cultured O/N with autologous sorted Tconv from PB at a ratio of 1:5 to induce antigen-specific activation. Afterward, the cells were washed with FACS buffer and treated with human Ig (Sandoglobulin, CSL Behring AG, PZN-0571760, 1.5 mg ml^−1^) to block unspecific Ab binding. After coating the cells with the IFNγ-catch reagent (cat. 130-054-202, Miltenyi Biotec, 1:10) for 5 min on ice, IFNγ secretion was induced during slow but continuous rotation at 37 °C for 45 min. Subsequently, the cells were stained with the PE-conjugated IFNγ detection Ab (cat. 130-054-202, Miltenyi Biotec, 1:10) in MACS buffer for 10 min on ice. In a second staining step, the cells were incubated with anti-CD4-APC-Cy7 (clone RPA-T4, BD Biosciences, 1:20) and anti-CD3-AmCyan (clone SK7, BD Biosciences, 3:20) in FACS buffer for 10 min on ice. Finally, cells were washed and resuspended in FACS buffer containing PI (BD Biosciences, 1:20) for dead-cell exclusion. MAMI- or IgG-specifically activated Tconv were analyzed using a Flow Cytometer and Cell Sorter FACSAria III (BD Biosciences) with FACSDiva software (version 6.1.3 and 8.0.1, BD Biosciences). For further TCRβ-specific cDNA amplification and sequencing viable IFNγ^+^CD3^+^CD4^+^Teff were sorted into PCR 8-well stripes (Nerbe) arranged in a 96-well format, one single-cell per well already containing SuperScript III Reverse Transcription buffer (Invitrogen), following a well-established single-cell sorting protocol^118^. In the end, single-cell sorted cells were frozen using liquid nitrogen and stored at –80 °C O/N until the initiation of single-cell RT-PCR.

### TCRβ CDR3 amplification and sequencing by single-cell multiplex RT-PCR

The TCRβ CDR3 sequence of individual single-cell sorted MAMI- or IgG-reactive IFNγ^+^Teff was amplified using a three-step single-cell mRNA-based multiplex PCR method^39^. In short, frozen single-cell sorted IFNγ^+^Teff were shortly boiled to induce cell lysis followed by Reverse transcription with a TCRβ-chain *TRBC*-specific primer that binds to the constant gene (*TRBC*) of the TCRβ-chain gene (Supplementary Table 5). Reverse Transcription was performed at a final volume of 15 µl, containing 1× First-Strand buffer (Invitrogen) with 0.5% (w/v) Triton X-100 (Sigma), 0.5 mM dNTPs (Invitrogen), 0.2 µM of the *TRBC*-specific reverse primer BCRT (Eurofins MWG Operon), 20 U RNaseOUT Recombinant RNase Inhibitor (Invitrogen), 100 µg ml^−1^ gelatin (Roche), and 200 U Superscript III Reverse Transcriptase (Invitrogen), at 50 °C for 90 min followed by enzyme denaturation at 95 °C for 5 min. This cDNA was then used as a template of a 1^st^ multiplex PCR reaction containing 24 forward TRBV primers, each specific for a different TCRβ-chain variable gene (*TRBV*) family, and one nested reverse *TRBC*-specific primer (Eurofins MWG Operon, Supplementary Table 5). The 1^st^ multiplex PCR reaction was performed in a total volume of 50 µl, including 10 µl single-cell cDNA, 1× PCR buffer (minus Mg, Invitrogen), 2 mM MgCl_2_ (Invitrogen), 0.25 mM dNTPs (Invitrogen), 5 nM of the nested *TRBC*-specific reverse primer 3BCRT, 24 *TRBV*-specific forward primers, 5 nM each, and 1.25 U of Platinum Taq DNA polymerase (Invitrogen). The 1^st^ multiplex PCR was run at 95 °C for 2 min followed by 40 cycles of denaturation at 94 °C for 45 s, primer hybridization at 57 °C for 45 s and DNA elongation at 72 °C for 50 s and consequently with a final elongation at 72 °C for 7 min. The 1^st^ PCR product was divided in 8 different 2^nd^ PCR reactions, each of which contained a group of 2, 3, or 5 TRBV forward primers and a second nested reverse *TRBC*-specific primer (Eurofins MWG Operon, Supplementary Table 5). 1 µl of the 1^st^ multiplex PCR product was distributed in 8 different tubes (A-H), each filled with 24 µl of 1×PCR buffer (minus Mg, Invitrogen), 2 mM MgCl_2_ (Invitrogen), 0.2 mM dNTPs (Invitrogen), 1 U Platinum Taq DNA polymerase (Invitrogen), 0.15 µM of the nested *TRBC*-specific reverse primer 5BCRT and 0.15 µM of each *TRBV*-specific forward primer from one out of eight possible primer sets (A-H, Supplementary Table 5). The 2^nd^ multiplex PCR was run at 95 °C for 2 min followed by 35 cycles of denaturation at 94 °C for 45 sec, primer hybridization at 57 °C for 45 s and DNA elongation at 72 °C for 40 s and consequently with a final elongation at 72 °C for 7 min. The 2^nd^ PCR products were run on a 2% agarose gel, and the ones that gave a band at ~350 base pairs (bp) were considered positive. For each positive 2^nd^ PCR product, we performed 2–5 new 3^rd^ PCR reactions with each TRBV forward primer separately and exactly as explained for the 2^nd^ multiplex PCR reaction step. Using DNA electrophoresis, we could identify the forward TRBV primer responsible for the TCRβ CDR3 amplification. In the end, each positive single-cell 3^rd^ PCR product was sequenced with the respective TRBV primer using Sanger Sequencing (GATC Biotech and LGC Genomics) and without previous purification. Raw sequencing data are available at the European Genome-Phenome Archive under the accession code EGAD00001004385.

### High-throughput RNA-based TCRβ sequencing

TCRβ high-throughput sequencing of RNA isolated from PB-derived total Tconv and total Treg populations was performed using unbiased PCR protocols^24,43,44,119^, independently of multiplex PCR reactions. As starting material we used RNA isolated from 8 × 10^3^ to 8.5 × 10^7^ cells (Supplementary Table 2), as described above. In the case of very low MAMI- and IgG-IFNγ^+^Teff cell numbers (patient 4573, 4578), we used cells sorted directly in tubes already containing Superscript III Reverse Transcription buffer (Invitrogen) and frozen at –80 °C O/N until the initiation of the Reverse Transcription step. Samples were sequenced using the MiSeq platform (Illumina), and raw reads were sorted according to the individual barcode combination used for each specimen. TCR sequence retrieval and annotation were obtained with an in-house-made pipeline used to trim and demultiplex the MiSEQ reads and the MiTCR software (version 1.0.3)^120^. The in-house-made pipeline is a customization of the code described in the GENEIS repository [https://github.com/G100DKFZ/gene-is]. More precisely, it includes only the first stage of the pipeline that is used to demultiplex the samples. Raw sequencing data can be accessed at the EGA database under the accession code EGAD00001004385.

### Isolation of blood-derived MAMI-specific and total tumor-infiltrating CD4^+^ TC using single-cell FACS sorting toward single-cell transcriptome sequencing

#### Isolation of tumor-infiltrating CD4^+^ TC

Tumor tissue was cut into small pieces submerged in PBS supplemented with benzonase (Merck, 100 U ml^−1^) and subsequently filtered through a 100-µm Falcon Cell Strainer (BD Biosciences, 352360) followed by centrifugation at 300×*g* for 10 min.

#### Isolation of blood-derived MAMI-specific CD4^+^ TC using the IFNγ-secretion assay

PB-derived Treg-depleted Tconv were stimulated with autologous MAMI-presenting DC and the IFNγ Secretion Assay–Detection Kit (PE) human (cat. 130-054-202, Miltenyi Biotec) was applied as described in detail above until the point of washing after staining with the anti-IFNγ-PE antibody alone.

#### Isolation of blood-derived MAMI-specific CD4^+^ TC using mam_34–48_ class II tetramers

Purified TC were washed and subsequently incubated for 2 h at 37 °C with 50 µl FACS buffer containing 6 μg ml^−1^ PE-labeled tetramers loaded either with mam_34–48_ or with a Class II-associated invariant chain (CLIP)-derived peptide.

The above cell suspensions were first treated with human Ig (Sandoglobulin, CSL Behring AG, PZN-0571760, 1.5 mg ml^−1^) in FACS buffer followed by cell-surface marker staining in MACS buffer. First, cell suspensions were incubated with anti-CD25-Viobright-FITC (clone 4E3, Miltenyi Biotec, 1:10) and anti-CD127-APC (clone MB15-18C9, Miltenyi Biotec, 1:10) for 10 min at 4–8 °C. After washing with MACS buffer, cells were further stained with CD3-AmCyan (clone SK7, BD Biosciences 1:20), CD4-V450 (clone RPA-T4, BD Biosciences, 1:20), and CD45RA-APC-H7 (clone HI100, BD Biosciences 1:20) for 20 min in ice. Consequently, the cells were washed, single-cell filtered, and resuspended in FACS buffer supplemented with Propidium Iodide (PI, BD Biosciences, 1:20) for dead-cell exclusion. Finally, the live CD4^+^ gate cells were designated conventional (Tconv, CD4^+^CD25^−^CD127^+/−^) regulatory (Treg, CD4^+^CD25^+^CD127^-^) or Activated Tconv (ActTconv, CD4^+^CD25^+^CD127^+^) and simultaneously ‘index’ FACS sorted using a Flow Cytometer and Cell Sorter FACSAria III (BD Biosciences) with FACSDiva software (version 8.0.1, BD Biosciences) directly into 2.5 µl lysis buffer RLT (Qiagen) supplemented with 1:20 SUPERase In (Ambion, 20 U µl^−1^)^55^ as one single cell per well in a FrameStar 96-well skirted PCR plate (4titude) and stored at −80 °C. To ensure high efficiency of single-cell deposition into the 2.5 µl volume of the lysis buffer, the cell sorter was adjusted separately before and after index sorting using a specially developed tool for visual control of single-cell deposition (further developed from reference^118^; S. Schmitt, personal communication).

### Single-cell poly(A) transcriptome sequencing

Single-cell transcriptome amplifications were performed on poly(A) transcripts contained within single-cell lysates that were captured with oligo-dT-coated beads (Dynabeads MyOne Streptavidin C1, Invitrogen) and amplified with a modified Smart-seq2 protocol adjusted on an automated liquid handling platform (Biomek FXP Laboratory Automation Workstation, Beckman Coulter; Sequencing Core Facility, Welcome Sanger Institute)^55,121^. The protocol was optimized using HD PB-derived Tconv and Treg at a resting state or after polyclonal stimulation for 48 h in anti-CD3 coated plates (clone OKT3, eBioscience, 0.5 µg ml^−1^,) with soluble anti-CD28 (clone CD28.2, eBioscience, 0.5 µg ml^−1^) (Supplementary Fig. 6a). Prior to reverse transcription, the External RNA Controls Consortium (ERCC, Ambion, 1:7,5 × 10^6^) was added to each reaction as spike-in controls. The amplified cDNA products were purified using Agencourt AMPure XP beads (Beckman Coulter) and resuspended in EB buffer (Qiagen). Libraries were generated using a modified Nextera protocol (Single-Cell Core Facility, Welcome Sanger Institute). Pooled 384-samples were sequenced aiming at an average depth of 1.3 million reads per well/single-cell using Illumina Hiseq V4 (pair-end 125-bp reads). Raw sequencing data can be accessed at the EGA database under the accession code EGAD00001004069.

### Single-cell transcriptome analyses

#### Pre-processing and quality control of single-cell RNA reads

Read pairs were trimmed for any adaptor contamination and low-quality reads were filtered using the following criteria: if >3 bases overlap with adaptor sequence, the overlapping sequence was trimmed off the 3′ end of any read. After adaptor trimming, both reads of a read pair should be >20 bases long, otherwise, they were discarded. Finally, the maximum allowed error rate was 0.1. Reads that passed the quality criteria were aligned to the human reference genome (hg19) using STAR (version 2.5.2)^122^ with default parameters. FeatureCount (version 1.5.1)^123^ was used to count the number of uniquely mapped reads located in each gene. Genes having a count of zero across all cells were removed from the count table.

#### Post-mapping quality control and scRNA-seq data analysis

Cells with less than 500 or over 10,000 genes expressed, >20% ERCC counts, and >15% mitochondrial counts were filtered. Across the whole dataset 1848 single cells passed the filters with overall 24,282 genes expressed. For the patient set post-filtering 1095 cells (break down of the number of cells per patient is as follows; MaCa 5: 323 cells, MaCa 7: 263 cells, MaCa 8: 189 cells, MaCa 9: 320 cells) passed the quality control filters with 19,279 features detected across all cells. For each SC3 (version 1.12.0)^124^ run, the consensus matrix was plotted, silhouette values were calculated and cluster-specific genes were identified. This information was used to determine the optimal k (number of clusters) and n (number of cells) values. To identify sub-clusters the above procedure was applied to each obtained cluster. The t-SNE method implemented in R (version 3.6.0) package Rtsne was used for cluster visualization. To validate the clustering results obtained from SC3, we also performed clustering analysis using Seurat (version 3.1.0)^125^. After removing low-quality cells from the dataset (mentioned above), we employed global-scaling normalization to normalize the feature expression measurements for each cell by the total expression, multiplied by 10,000 scale factors, followed by log2 transformation. To identify genes that exhibit high cell-to-cell variation in the dataset, the correlation between mean expression and dispersion was fitted to the logR values (variance to mean ratio). The dispersion cut-off was set at 0.5. Next, we performed PCA on the scaled data. Significant components were selected by resampling test to reconstruct a null distribution and significant PCs with low p values were selected. K-nearest neighbor (KNN) test was used to identify the number of clusters. t-SNE was used for nonlinear dimensional reduction using the significant PCs identified above. Overlapping genes between the two methods were selected as differentially expressed genes per cluster. We performed a pairwise comparison between different clusters using DEseq2^126^ in R (version 3.5.1) with R package (DESeq2_1.20.0). The matrix of feature counts was loaded within R and cells with ERCC counts of at most 20, mitochondrial counts <15%, and total features >500 and <10,000 were retained. Two-class differential expression analysis with the function DESeq with default parameters was run to perform a pairwise comparison between different clusters. This performs negative-binomial generalized linear model fitting and uses Wald tests for significance. The code used for single-cell transcriptome data analysis is described in Supplementary Software 1.

#### Pseudotime analysis

To identify diversity and lineage differentiation between Tconv, ActTconv, and Treg labeled cells from breast tumors, we applied the Monocle2 algorithm (version 2.12.0)^127^. The CellData set object was created with the following parameters; lowerDetectionLimit = 0.5 and expressionFamily = negbinomial.size. For feature selection, we used an unsupervised approach. Reversed graph embedding^128^ (DDRTree) was used to reduce the data’s dimensionality based on selected features. The CD4^+^ TC differentiation trajectory was inferred after dimension reduction and cell ordering with the default parameters of R package Monocle. To confirm the trajectory obtained by the Monocle2 algorithm, we also analyzed tumor-infiltrating Tconv, ActTconv, and Treg using the Slingshot (version 1.2.0) pseudotime method^129^. Filtered counts were normalized using the Seurat R library, and the second set of pseudotime measurements were estimated using the Slingshot library^129^. Using Slingshot the likely lineage structure was identified based on the cluster information generated from Seurat (SLM clustering algorithm). The code used for single-cell transcriptome data pseudotime analysis is described in Supplementary Software 2.

#### TCRαβ nucleotide sequence gene analysis from single-cell RNA sequencing data

We use TraCeR^56^ (version commit 4346db1e2cd88ec3551069066a1af1e152897c08” from 2017-05-15 in-between official release versions v0.5.1 and v0.6.0) in mode “assemble” to reconstruct TCR sequences from single-cell RNA-seq reads. In order to speed up the transcript expression quantification involved in the reconstruction process, we implemented some modifications to the original TraCeR workflow (resulting in speed-up factors of up to 4). These modifications have been made publicly available in TraCeR through the option “--small_index” (see https://github.com/Teichlab/tracer). To determine TCR-clonotype clusters, we use the full “unfiltered” output created by TraCeR for each individual cell and select only those reconstructed TCR sequences which are determined to be productive for further analysis. These, we then split into sets *S*^*(A)*^ and *S*^(*B*)^ of TCRα and TCRβ sequences, respectively. Next, we define our own TPM-based filtering in the following way: For a set *S* of *N* sequences, $S = \left\{ {S_i} \right\}_{1 \le i \le N}$, with corresponding TPM values *T*_*i*_, we order the set such that *T*_*i*_ ≥ *T*_*j*_ for *i* < *j* and afterwards determine the minimal value of *M* such that $\mathop {\sum}\nolimits_{i = 1}^M {T_i \ge h} \cdot \mathop {\sum}\nolimits_{i = 1}^N {T_i}$ with threshold *h* set to *h* = 0.95. Our filter function *F*_*h*_ is then defined as $F_h\left( S \right) = \left\{ {S_i} \right\}_{1 \le i \le M}$. This means that the sequences with lowest corresponding TPM values are ignored, as long as the sum of their TPM values is smaller than 5% of the sum of all TPM values (corresponding to all the sequences in the original set *S*). Note that the filter has no effect if the smallest TPM value is larger than 5% of the sum of all TPM values. Based on this filtering, we then define four different equivalence relations between single cells:

1. Two cells are defined to be equivalent if $F_h\left( {S^{\left( A \right)}} \right)$ yields completely identical sets of sequences (containing at least one sequence) for both cells. This means all TCRβ sequences are ignored, as well as TCRα sequences with low TPM values (as described above).
2. Two cells are defined to be equivalent if $F_h\left( {S^{\left( B \right)}} \right)$ yields completely identical sets of sequences (containing at least one sequence) for both cells. This means all TCRα sequences are ignored, as well as TCRβ sequences with low TPM values (as described above).
3. Two cells are defined to be equivalent if $F_h\left( {S^{\left( A \right)}} \right) \cup F_h\left( {S^{\left( B \right)}} \right)$ yields completely identical sets of sequences (containing at least one sequence in *S*^(*A*)^ or *S*^(*B*)^) for both cells. This means that the filtering is applied to TCRα and TCRβ sequences separately. Note that one of the sets (*S*^(*A*)^ or *S*^(*B*)^) may be empty for both cells.
4. Two cells are defined to be equivalent if they are equivalent according to (1) and (2) at the same time. This means that the TPM-based filter is applied to TCRα and TCRβ sequences separately and that cells have to have at least one productive TCRα sequence and one productive TCRβ sequence.

In all of the above equivalence relations, two individual TCR sequences are identified as identical if their junctional sequences as well as their assigned V-, J-, and D-gene segments are identical (not discriminating between different allele versions). We define our TCR-clonotype clusters as equivalence classes according to one of the equivalence relations (1) through (4). Note that by definition each element of an equivalence class is equivalent to each other element of the same class.

For our final results, we used option (4) restricted to cells with at most two productive TCRβ and two productive TCRα sequences (after filtering with *F*_*h*_). Using this method, we obtained a total of 45 TCR-clonotype clusters with

- 39 clusters being defined by one TCRα and one TCRβ sequence,
- 4 clusters being defined by two TCRα and one TCRβ sequence,
- 2 clusters being defined by one TCRα and two TCRβ sequences.

### Triple immunofluorescent staining of tumor-infiltrating CD4^+^ subsets

FFPE breast tumor tissue was cut into 10-µm sections for single-cell laser microdissection (SC-LMD) or 20-µm sections for Confocal Microscopy (CM) using an automatic microtome (HM 355S, Thermo Scientific). CM-Tissue sections were mounted on glass microscope slides (Thermo Scientific). In the case of SC-LMD, tissue sections were mounted on FLUO-Membranes (cat. 11600250, Leica Microsystems), which had been previously treated with UV light for 30 min followed by washing with Acetone (AppliChem) for 10 s, then with 8% (3-Aminopropyl) triethoxysilane (APES, Sigma-Aldrich) in Acetone for 3 min, twice more with Acetone and finally with dH_2_O for 10 min before drying at 37 °C O/N. Subsequently, tissue sections were dried first at room temperature for 1 h and then at 60 °C O/N. To induce deparaffinization, tissue sections were transferred thrice for 5 min in Xylol (Medite) followed by 2 min incubation in 99% ethanol (Medite), then in 96% ethanol, and finally in 70% ethanol followed by immersion into TBS buffer (0.8% NaCl (Sigma), 0.02% KCl (AppliChem) 0.3% Tris (Roth) in dH_2_O, pH = 7.4). Antigen retrieval was performed by heating in EDTA solution (pH = 9) for CM or in Bond Epitope Retrieval Solution 2 (Leica Biosystems) for SC-LMD for 20 min at 95 °C using a water bath (Gesellschaft für Labortechnik mbH) with subsequent cooling down for 20 min at room temperature. From this point, the sections were washed three times for 5 min in TBS at room temperature after every step of the staining protocol. To block unspecific binding of the antibodies, tissue sections were treated with 10% goat serum (cat. S-1000, LINARIS Biological products GmbH) in PBS (Biochrome AG) for 15 min (SC-LMD) or 30 min (CM) at room temperature. Tissue sections were fluorescently labeled in five sequential steps. First, they were incubated with rabbit anti-IL7R a chain (polyclonal, cat. ab115249, Abcam, 1DB_ID:1DB-001-0001137020, 1:100 for SC-LMD or 1:200 for CM) and mouse anti-CD4 (clone 4B12, cat. NCL-L-CD4-368, Novocastra, 1:50 for SC-LMD or 1:30 for CM) in PBT buffer (5% BSA (Roth), 0.5% Tween20 (Calbiochem), 0.02% NaN_3_ (AppliChem) in PBS (Sigma-Aldrich)) at 4 °C O/N. Second, they were stained with goat anti-rabbit IgG(H + L) Alexa 647 (polyclonal, cat. A-21244, Invitrogen, 1:100) and goat anti-mouse IgG(H + L) Alexa 488 (polyclonal, cat. A-11029, Invitrogen, 1:100) at Room Temperature for 1 h. In a third step tissue sections were treated with mouse anti-FOXP3 (clone 236A/E7, cat. 14-4777, eBioscience, 1:50 for SC-LMD or 1:30 for CM) for 2 h at Room Temperature followed by labeling with donkey anti-mouse IgG(H + L) Alexa 594 (polyclonal, cat. A-21203, Life technologies, 1:100) in PBT for 1 h at room temperature. Nuclear DNA was labeled using 1 µg ml^−1^ DAPI (AppliChem) in PBS. CM-sections were covered with vectashield mounting medium (Vector Laboratories) and cover glass to prevent the photobleaching and kept at 4 °C. SC-LMD sections were immersed in 1% glycerol (AppliChem) in PBS and after drying they were maintained at 4 °C.

### Confocal Microscopy of tumor-infiltrating CD4^+^ subsets

The CM-stained sections were scanned with Leica TCS SP5 DMI6000 confocal microscope (Leica microsystems) and Leica LAS AF software (data acquisition and analysis, version 1.8.2, Leica microsystems) using 63x glycerol immersion objective at a pinhole diameter of 1 “Airy unit”. DAPI, Alexa Fluor 488, Alexa Fluor 594, and Alexa Fluor 647 dyes were excited by laser lines 405, 488, 550, and 633 nm, respectively. The Leica TCS SP5 DMI6000 is equipped with tunable spectral detection windows for its photo-multiplier tubes (PMT) and the detection ranges were set as follows: DAPI was detected at a range of 414–478 nm, Alexa Fluor 488 at a range of 504–582 nm, Alexa Fluor 594 at a range of 620–650, and the Alexa Fluor 647 at a range of 648–736 nm. To avoid any chance of cross-talk between the signals, the “sequential scan” feature of the microscope was used. The DAPI channel was collected together with Alexa Fluor 594 channel, and Alexa Fluor 488 together with Alexa Fluor 647, noting there was no observed cross-talk between the simultaneously collected channels. Samples were imaged at a zoom factor of 2, resulting in a field size of 119 × 119 µm. With 1024 × 1024 pixels, the resulting pixel size in the *xy* plane was 116 × 116 nm. The distance between neighboring *xy* images was set to 122 nm. The stack images were generated using open-source ImageJ software (version 1.47i, Wayne Rasband, National Institute of Health, USA).

### Single-cell laser microdissection of tumor-infiltrating CD4^+^ subsets

Fluorescently labeled tumor-infiltrating TT Treg, TT Tconv, and TT ActTconv were cut from FFPE tissue sections by single-cell laser microdissection using the LMD7000 system (Leica microsystems). To improve tissue morphology during cutting, the tissue section was briefly immersed in 70% ethanol (Carl Roth GmbH & Co. KG) just before single-cell cutting. Labeled tissue sections were observed under a fully automated upright Leica DM6000 B microscope (Leica Microsystems) with a ×63 objective in combination with a Leica DFC365 FX camera using the Leica Laser Microdissection LMD 7.4.1.4815 software (Leica microsystems). Single cells were identified on the basis of nuclei staining with DAPI and were further analyzed for positive/negative expression of CD4-Alexa 488, FOXP3-Alexa 594, and CD127-Alexa 647 using the LDA, LMG, LA5, and LC5 filter, respectively. Accordingly, CD4^+^ cells were grouped in three different subsets: FOXP3^+^CD127^−^ TT Treg, FOXP3^-^CD127^+^ TT Tconv and FOXP3^+^CD127^+^ TT ActTconv. Single cells were cut and carefully separated from their neighboring tissue and cells using a Laser Beam. Simultaneously, cells of the same group were collected driven by gravity on the respective flat cap of a 0.2-ml Eppendorf tube. At the end of each day, the collected cells were centrifuged for 15 min at 4 °C at full speed and stored at –20 °C until gDNA extraction.

### Isolation of gDNA from tumor-infiltrating CD4^+^ subsets

The extraction of gDNA from laser-microdissected breast tumor-infiltrating TT Treg, TT Tconv, and TT ActTconv was performed using the QIAamp DNA Micro Kit (Qiagen) as suggested by the manufacturer with slight modifications. Eppendorf tubes containing up to ~400 microdissected cells of the same subset were transferred from –20 °C directly into a table centrifuge for a short spin down. Cell lysis was induced by the addition of ATL buffer (Qiagen) followed by incubation with Proteinase K (Qiagen) at 56 °C for 16 h with occasional agitation. Due to the small total number (863–1920) of microdissected cells collected per subset for each patient, AL buffer was mixed with carrier RNA (Qiagen), while lysates derived from the same CD4^+^ subset were pooled before loading on the QIAamp MinElute column (Qiagen). Finally, gDNA was extracted using 40 µl AE buffer (Qiagen) and stored at –20 °C until sequencing.

### High-throughput gDNA-based TCRβ sequencing of tumor-infiltrating CD4^+^ subsets

TCRβ high-throughput sequencing of gDNA isolated from tumor-infiltrating laser-microdissected TT Tconv, TT Treg, TT ActTconv, and from TT Total TC (gDNA isolated from ten additional serial 25 µm FFPE TT sections) of breast cancer patients was performed using the ImmunoSeq human TCR-β assay at survey level by Adaptive Biotechnologies Corp. (Seattle WA, USA)^108^. The respective sequencing data are provided at the EGA database under the accession code EGAD00001006428.

### Nucleotide-amino acid sequence data analysis

The analysis of TCRβ nucleotide/amino acid sequences obtained by Sanger sequencing of PCR products from single-cell sorted IFNγ^+^Teff was performed using the DNASTAR Lasergene software package (version 7, DNASTAR). To determine the CDR3 sequence and the TRBV/TRBJ usage of each retrieved TCRβ chain, we applied the IMTG VQUEST (version 3.2.23, IMTG, University of Montpellier) and the IMTG Junctional Analysis Tool (version 2.1.0, IMTG, University of Montpellier) provided online. Characterization and analysis of TCRβ sequences resulting from gDNA-based high-throughput sequencing of breast tumor-infiltrating TT Tconv, TT Treg, TT ActTconv, or TT Total TC were performed using the immunoSEQ Analyzer platform (version 2.0, Adaptive Biotechnologies). Multiple Sequence alignment of TCRβ nucleotide sequences was performed with the help of ClustalW2-Multiple Sequence Alignment software (version 2.1, EBI, Cambridge). Pie charts, Venn diagrams, and clonal frequency distribution graphs were performed using Microsoft Excel (version 2013, Microsoft, USA) and GraphPad Prism (version 8.0.2, GraphPad Software, La Jolla, CA, USA).

### Statistical analysis

The degree of similarity between the different TCRβ repertoires was assessed using the Morisita–Horn (MH) similarity index^45^. The MH-index ranges from 0 to 1, where 0 represents no similarity, while 1 corresponds to completely identical populations. Most importantly, the MH index considers not only the number of shared TCR sequences between two different TCR repertoires but also their contribution to each TCR repertoire. To investigate whether the frequencies of overlapping clones between Treg and Tconv or antigen-specific IFNγ^+^Teff exceed the rate, which can be expected due to contamination during the separation process, exact two-sided 95%-Pearson–Clopper confidence intervals were calculated for the frequency of Treg in the Tconv subset and the proportion of Tconv in the Treg subset. To estimate if one missed a relevant clone in either Treg, Tconv, Teff, or ActTconv, one-sided 95%-Pearson–Clopper intervals for an observed frequency of zero were calculated^130^. On this basis, it can be concluded with 95%-confidence that the frequency of missed clones does not exceed the value given by the upper limit of this interval (*φ*_max_)^130^. Considering that the PB obtained per patient was relatively small in comparison to the subject´s entire blood volume, the use of the binomial distribution instead of the hypergeometric distribution is justified. Due to great dissimilarities in the clone frequency distribution of Treg and Tconv across different individuals, the mean clonal frequency is not adequate to compare TCR diversity among Treg or Tconv between all tested healthy donors and tumor patients. Instead, the number of clones covering the upper 25% of the respective clonal distributions was used as a parameter, as a more meaningful way to depict and compare the clonal frequency of the most dominant clones per population and compare the TCR repertoires of different sample groups. Statistical comparison was performed by two-sided *t* test on rank data. To compare statistically Treg or Tconv between three MaCa patients and three HDs or two age-matched HDs, we transformed the original data to rank data (rank all five values and give rank values from 1 to 5) and then applied the *t* test to the rank data. The reason we performed this t-test and not the permutation test, is that the latter needs by construction at least three subjects per group to have the possibility to reach the 5% significance level. The same is true for the exact Wilcoxon rank-sum test, while the *t* test on the original data with equal variances is not justified at all. The Morisita–Horn index was calculated using the EstimateS package (version 9. beta 4, Robert K. Colwell, University of Connecticut, USA), while the estimation of frequencies including Pearson–Clopper confidence intervals was carried out using the Statistical Analysis System (Version 9.3, SAS Institute Inc, Cary, NC, USA).

## Supplementary information

Supplementary Information

Description of Additional Supplementary Files

Supplementary Software 1

Supplementary Software 2

## Data Availability

Raw sequencing data that support the findings of this study have been deposited in European Genome-Phenome Archive under the accession codes EGAD00001004385, EGAD00001004069, EGAD00001006428, and are available on application to the linked Data Access Committee upon request to Dr. Florian Schuetz at florian.schuetz@med.uni-heidelberg.de or florian.schuetz@diakonissen.de. Most additional raw data supporting the findings of this study are available within the paper and its supplementary information files. The remaining data are available from the corresponding author upon reasonable request. Source data are provided with this paper.

## Source data

Source Data

## Code availability

The code used for single-cell transcriptome data analysis is described in Supplementary Software 1 and for the pseudotime analysis in Supplementary Software 2.
