# Supplementary material for: Common clonal origin of conventional T cells and induced regulatory T cells in breast cancer patients
Source: Nat Commun. 2021 Feb 18;12:1119. doi: 10.1038/s41467-021-21297-y (PMC7893042; doi:10.1038/s41467-021-21297-y)
Supplement: Supplementary file 1 — Supplementary Information [file 41467_2021_21297_MOESM1_ESM.pdf]

Supplementary Information

Common clonal origin of conventional T cells and induced regulatory T cells in breast cancer patients

Maria Xydia[1,2]*, Raheleh Rahbari[3], Eliana Ruggiero[4], Iain Macaulay[3,5], Maxime Tarabichi[3,6], Robert Lohmayer[1,7], Stefan Wilkening[4], Tillmann Michels[1], Daniel Brown[8,14], Sebastian Vanuytven[6,8], Svetlana Mastitskaya[9,15], Sean Laidlaw[3], Niels Grabe[9,10], Maria Pritsch[2], Raffaele Fronza[4], Klaus Hexel[11], Steffen Schmitt[11], Michael Müller-Steinhardt[12], Niels Halama[9,10], Christoph Domschke[13], Manfred Schmidt[4], Christof von Kalle[4,16], Florian Schütz[13], Thierry Voet[3,8] and Philipp Beckhove[1,2]*

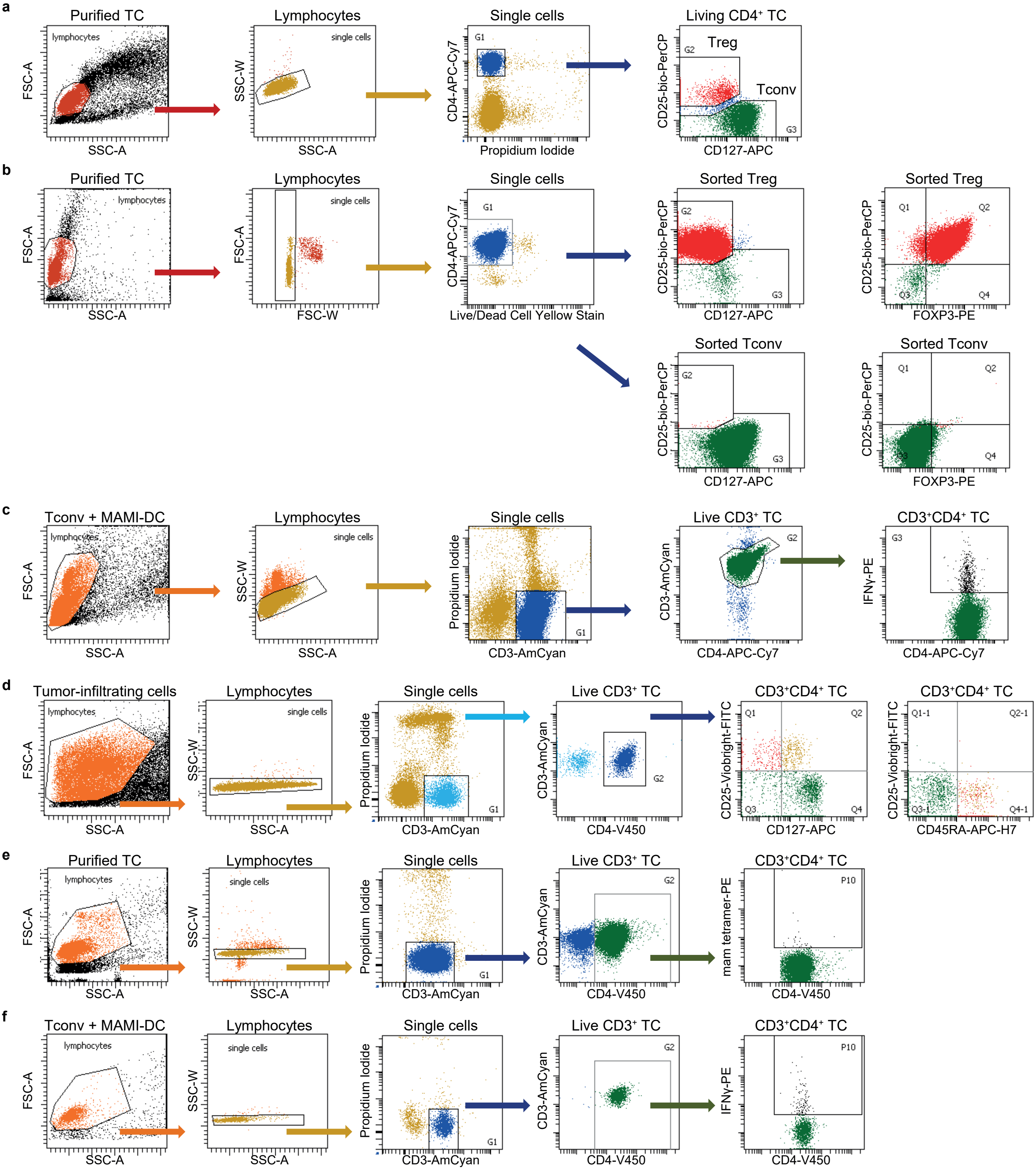

Supplementary Figure 1. Gating strategies used for FACS cell sorting and analysis. (**a**) Gating strategy to sort CD25^{+}CD127$^{-/low}$ Treg and CD25^{-}CD127$^{+/-}$ Tconv CD4^{+} cells from purified TC obtained from peripheral blood (PB) of Healthy Donors and breast cancer patients with mammary gland adenocarcinoma (MaCa) presented on Fig. 1a, b. (**b**) Gating strategy to analyze the purity of sorted Treg and Tconv on the basis of CD25, CD127 and FOXP3 expression depicted in Supplementary Fig. 2a. (**c, f**) Gating strategy to sort MAMI/IgG-reactive IFNγ^{+}Teff from sorted Tconv stimulated with MAMI/IgG-loaded autologous Dendritic Cells (DC) presented in Fig. 1c (**c**) and Supplementary Fig. 5d (**f**). (**d**) Gating strategy to sort Tconv Treg and ActTconv from breast tumor-infiltrating CD4^{+} TC in MaCa patients presented in Fig. 3a and Supplementary Fig. 5a, b. (**e**) Gating strategy to sort mam$_{34-48}$/CLIP-tetramer positive CD4^{+} TC from PB of MaCa patients presented in Supplementary Fig. 5c. G; Gate.

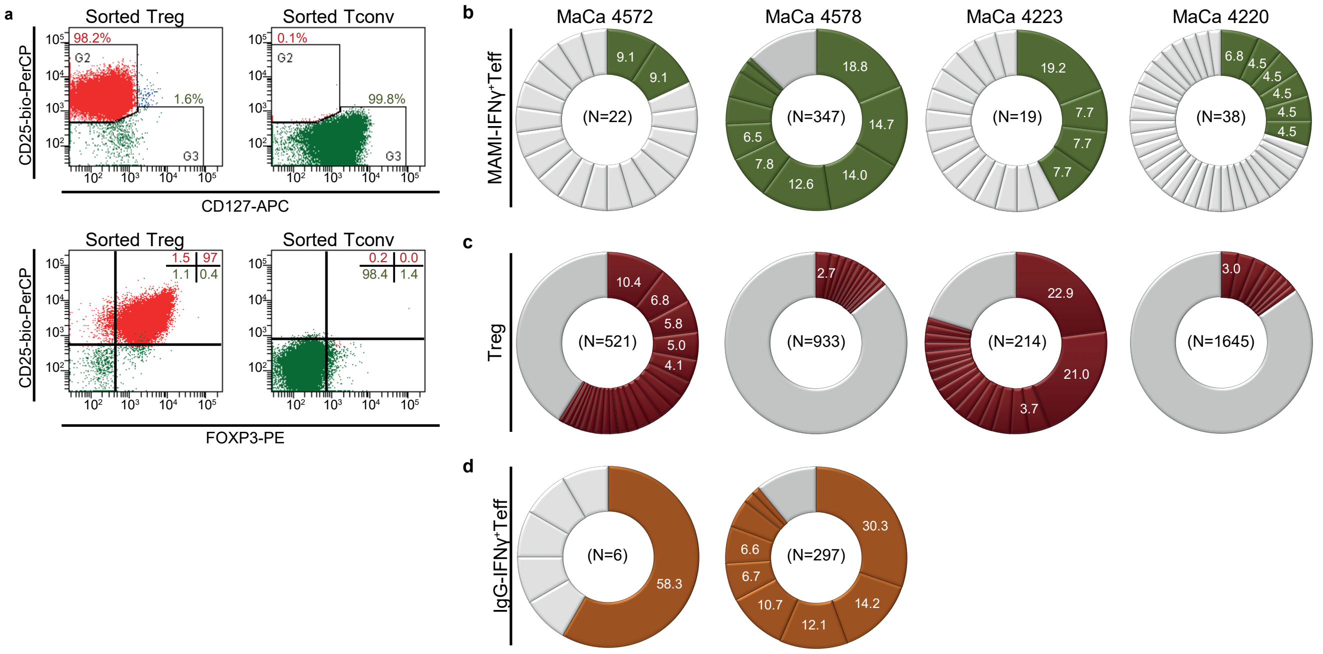

Supplementary Figure 2. Oligoclonal expansion of TAA-reactive Teff and total Treg in peripheral blood of breast cancer patients. (**a**) After-sorting purity of sorted Tconv and Treg populations based on surface CD25, CD127 and intracellular FOXP3 expression. FACS analysis of CD25 versus CD127 or CD25 versus FOXP3 expression on sorted Treg and Tconv populations. Representative data from peripheral blood (PB) of one healthy individual (n=3 biologically independent replicates). Percentages (%) indicate the frequency of Treg (G2, red) and Tconv (G3, green) in the total $CD4^+$ population analyzed per plot. G; Gate. (**b**, **c**, **d**) Pie chart frequency distribution of all clonotypes (unique TCRβ nucleotide sequences) obtained from MAMI-IFNγ^+Teff (**b,** green), Treg (**c**, red) and IgG-IFNγ^+Teff (**d**, orange) in PB of breast cancer patients with mammary gland adenocarcinoma (MaCa), presented as one pie-slice per clonotype clockwise in order of decreasing percentage (%, white). TCRβ sequences were characterized either by single-cell sorting and multiplex RT-PCR[1] (**b**, **d**, patient 4572, 4223, 4220) or high-throughput RNA-based sequencing of cells directly sorted into Reverse transcription buffer[2,3,4](**b**, **d**, patient 4578) and bulk RNA isolated from total Treg (**c**). In contrast to colorful pie slices, white slices represent clonotypes observed in only one cell (**b**, **d**, patient 4572, 4223, 4220) and grey slices represent the TCRβ repertoire fraction that contains clonotypes with frequency below 1% (**b**, MaCa 4578; **c**, all patients; **d**, MaCa 4578). N, total number of clonotypes. (**b, c**) n=5, (**d**) n=3. n refers to biologically independent replicates. Source data are provided as a Source Data file.

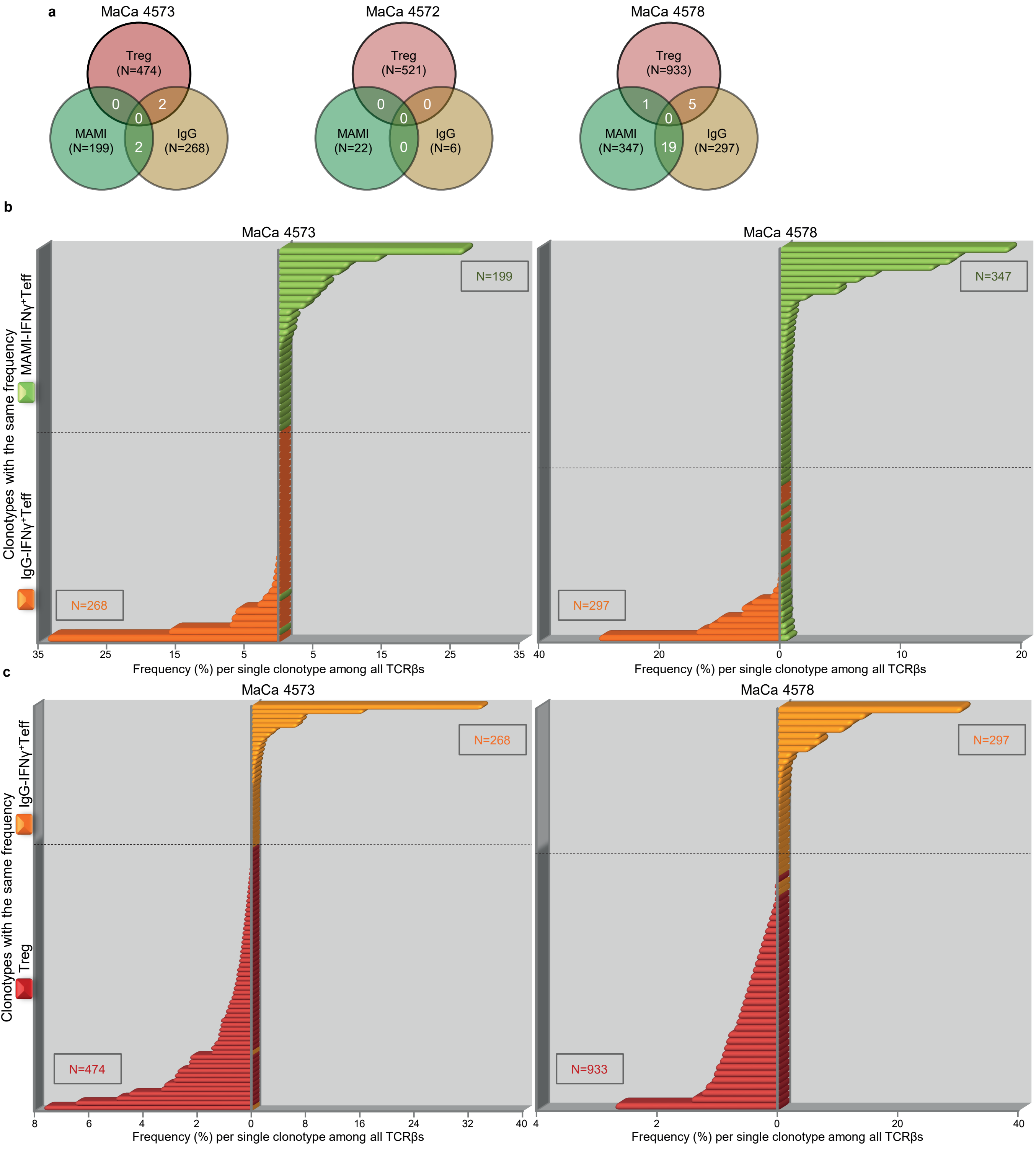

Supplementary Figure 3. No TCRβ similarity between MAMI- and IgG-responding IFNγ-secreting Teff versus total Treg in peripheral blood of breast cancer patients. (**a**) Venn diagram showing the number of common (white) clonotypes (unique TCRβ nucleotide sequences) shared between MAMI- and IgG-IFNγ$^+$Teff compared to total Treg within the same breast cancer patient with mammary gland adenocarcinoma (MaCa). (**b**, **c**) Frequency comparison of overlapping versus non-overlapping clonotypes between IgG-IFNγ$^+$Teff and MAMI-IFNγ$^+$Teff (**b**) or total Treg (**c**) in MaCa 4573 and 4578. Due to the big number of clonotypes obtained per subset, clonotypes are organized in groups, each with clones of identical frequency. Each bar represents a group (y axis) plotted against the frequency of each single clone per group (x axis). Clonotypes are arranged in order of decreasing (**b**, IgG-IFNγ$^+$Teff, orange; **c**, Treg, red) or increasing (**b**, MAMI-IFNγ$^+$Teff, green; **c**, IgG-IFNγ$^+$Teff, orange) frequency. Clonotypes derived from distinct subsets are separated by a dashed black line, while in case of overlapping clones between different subsets an orange and a green bar (**b**) or a red and an orange bar (**c**) align on the y axis. N, total number of clonotypes identified among MAMI-IFNγ$^+$Teff (green), IgG-IFNγ$^+$Teff (orange) and total Treg (red). n=3 biologically independent replicates. Source data are provided as a Source Data file.

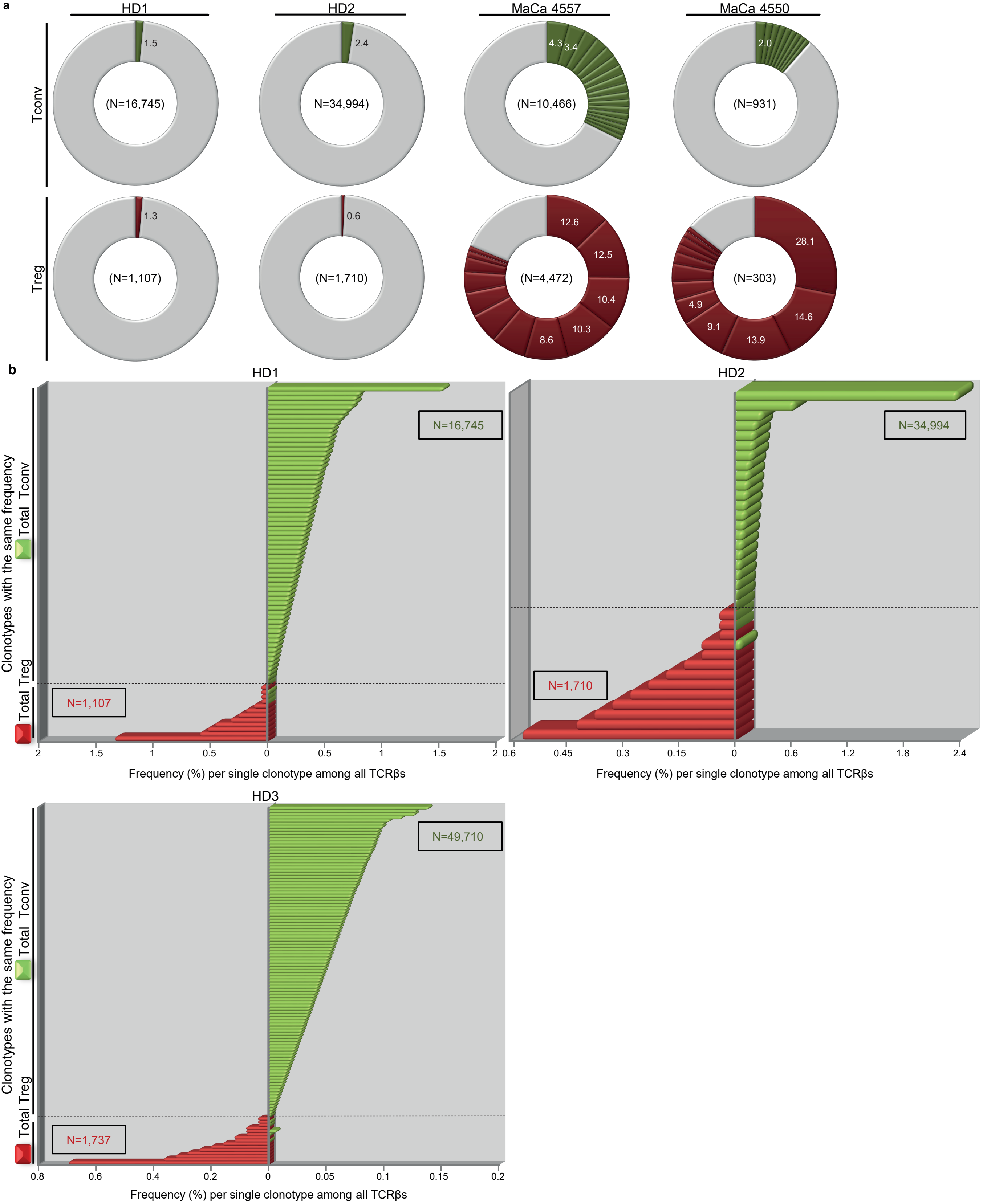

Supplementary Figure 4. Minor overlap between total Treg and Tconv in peripheral blood of breast cancer patients and healthy individuals. (**a**) Frequency distribution of all clonotypes (unique TCRβ nucleotide sequences) identified per subset depicted in a clockwise pie chart, one pie-slice per clonotype, in order of decreasing percentage (%) among total Tconv (green) and total Treg (red) from two breast cancer patients with mammary gland adenocarcinoma (MaCa, n=3) and two healthy donors (HD, n=3), n refers to biologically independent replicates. N, total number of clonotypes recovered per subset in each individual. Grey slices represent the TCRβ repertoire fraction that contains clonotypes with frequency below 1%. (**b**) Bar graph representation of the frequency of overlapping clonotypes (unique TCRβ nucleotide sequences) between total Tconv and Treg in relation to the frequencies of all clonotypes per subset in peripheral blood (PB) of HDs. Each column corresponds to a group of clones with the same frequency (y axis) and its height represents the frequency of a single clone in this group (x axis). Clonotypes are depicted in order of decreasing (Treg, red) and increasing (Tconv, green) frequency within each subset. Clonotypes derived from distinct subsets are separated by a dashed black line, while in case of overlapping clones between total Tconv and total Treg a green and a red bar align on the y axis. N, total number of clonotypes in Treg (red) or Tconv (green). n=3 HDs, n refers to biologically independent replicates. Source data are provided as a Source Data file.

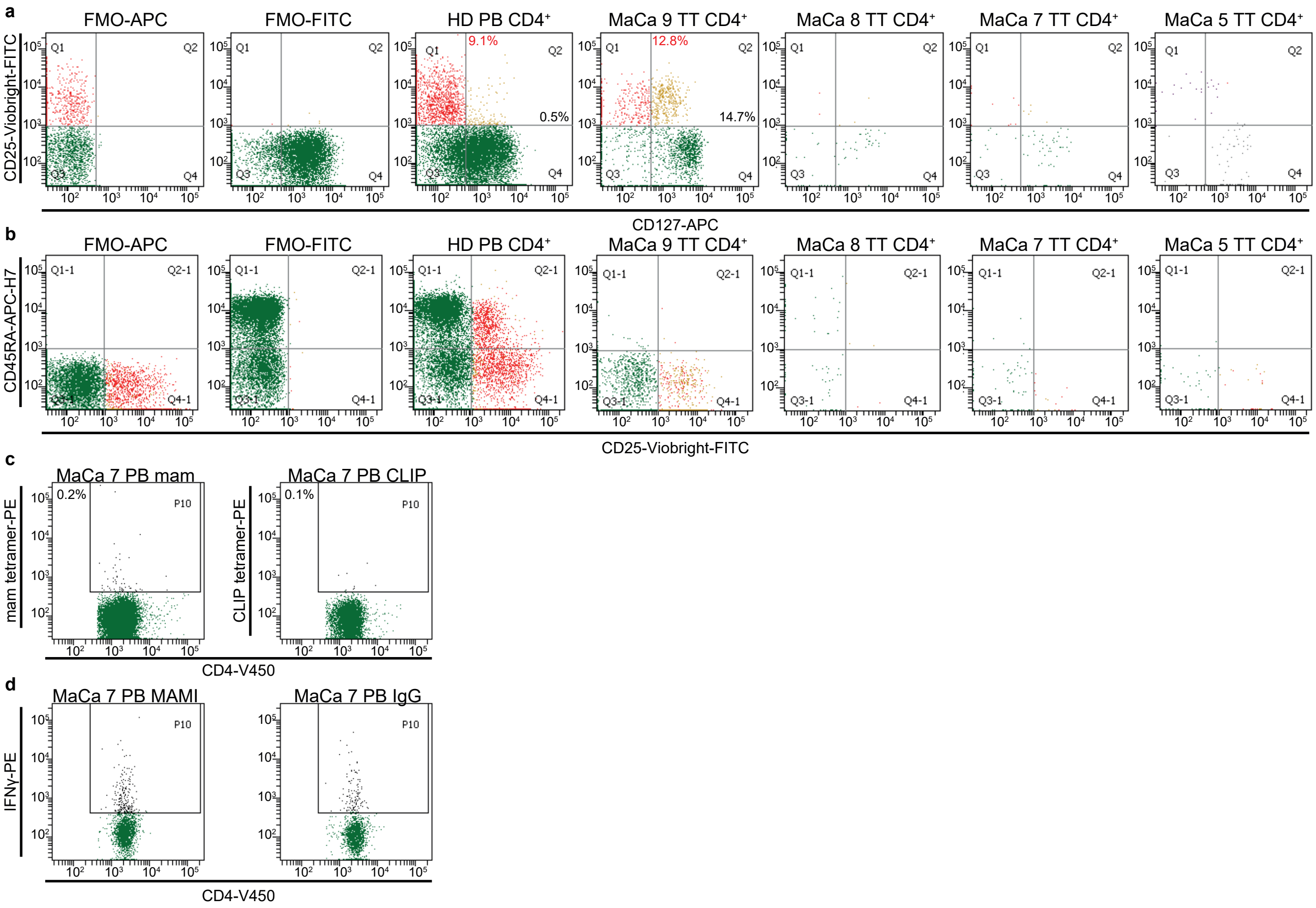

Supplementary Figure 5. Phenotypic characterization of circulating and tumor-infiltrating CD4⁺ TC in breast cancer patients. FACS analysis of CD25 versus CD127 (**a**) and CD25 versus CD45RA (**b**) cell surface expression on CD4⁺ TC from peripheral blood (PB) of a representative healthy donor (HD) and tumor tissue (TT) from 4 breast cancer patients with mammary gland adenocarcinoma (MaCa). (**a**) Percentages (%) indicate the frequency of Treg (Q1, red), ActTconv (Q2, orange) and Tconv (Q3 and Q4, green) in the total CD4⁺ population analyzed per plot. FMO, Fluorescence Minus One control after staining with fluorescent Abs against all markers apart from the one detected in the indicated fluorescent channel. (**c, d**) Isolation of TAA-specific CD4⁺ TC from PB of MaCa patients using FACS sorting. Frequency of $mam_{34\text{-}48}$/CLIP-tetramer positive CD4⁺ TC (**c**) and MAMI-reactive CD4⁺Teff based on IFNγ-catch assay (**d**) and FACS analysis. MaCa: n=4 HD: n=2, n refers to biologically independent replicates.

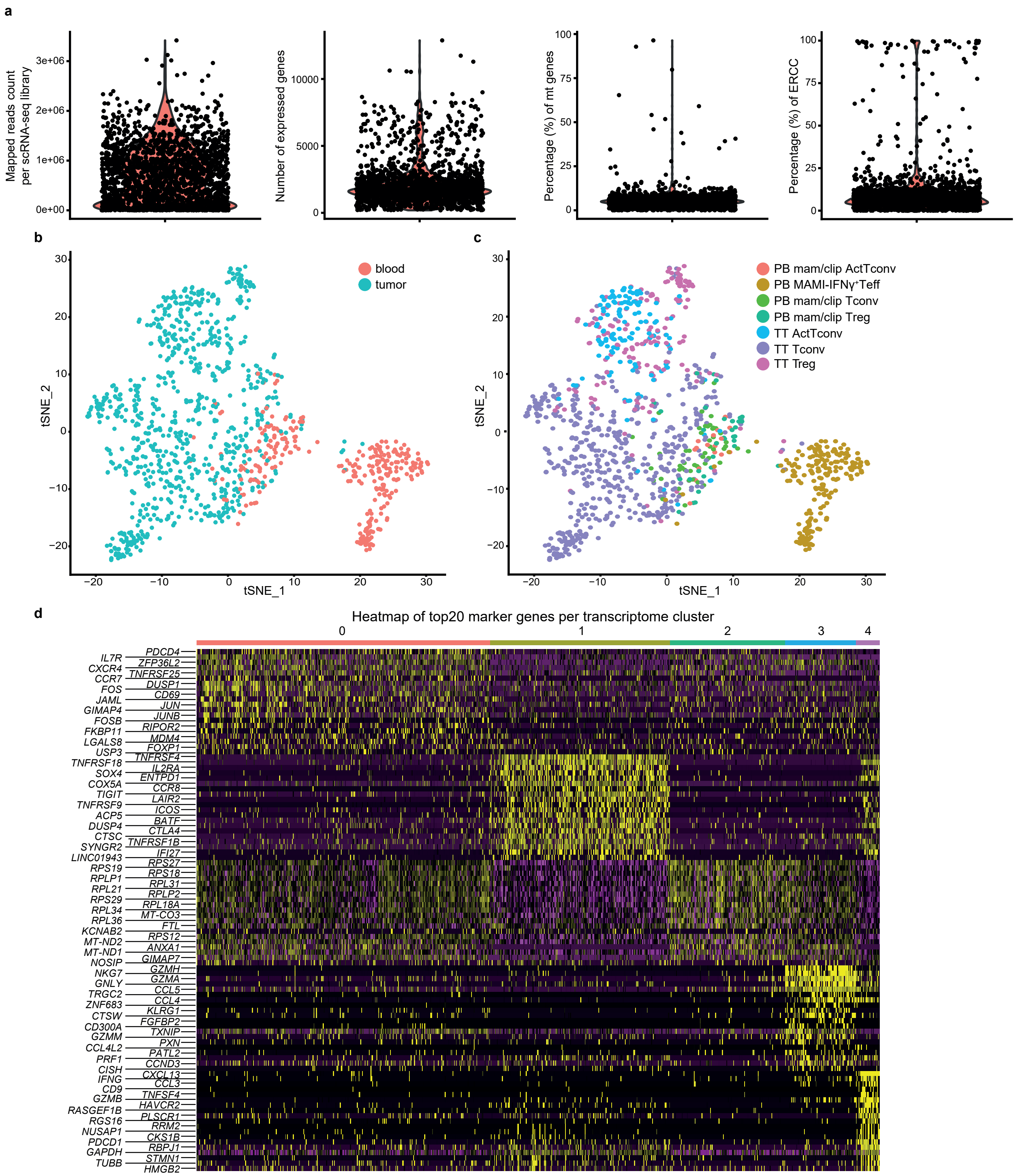

Supplementary Figure 6. Single-cell transcriptome sequencing in blood and tumor of breast cancer patients. (**a**) Quality control analysis of single-cell transcriptome data presented as Violin plot of the retrieved mapped reads per single-cell RNA-seq library, total number of expressed genes per single-cell, percentage of reads mapping to mitochondrial genes or ERCC spike-in sequences among total sequencing reads per single-cell. Single-cell transcriptome data from blood-derived and tumor-tissue (TT) infiltrating CD4$^+$ TC from all tested breast cancer patients (n=4 biologically independent replicates) and resting or anti-CD3/CD28 stimulated Tconv and Treg isolated from peripheral blood (PB) of n=1 healthy individual used for protocol establishment. (**b**, **c**) t-SNE representation of single-cell transcriptome data from TT-infiltrating CD4$^+$ TC and PB-derived MAMI-specific CD4$^+$ TC from n=4 breast cancer patients colored according to tissue origin (**b**) or cell surface phenotype (**c**). n, biologically independent replicates (**d**) Heatmap of the top 20 gene markers per transcriptome cluster among TT-infiltrating CD4$^+$ TC with high (yellow), intermediate (black) and no expression (purple).

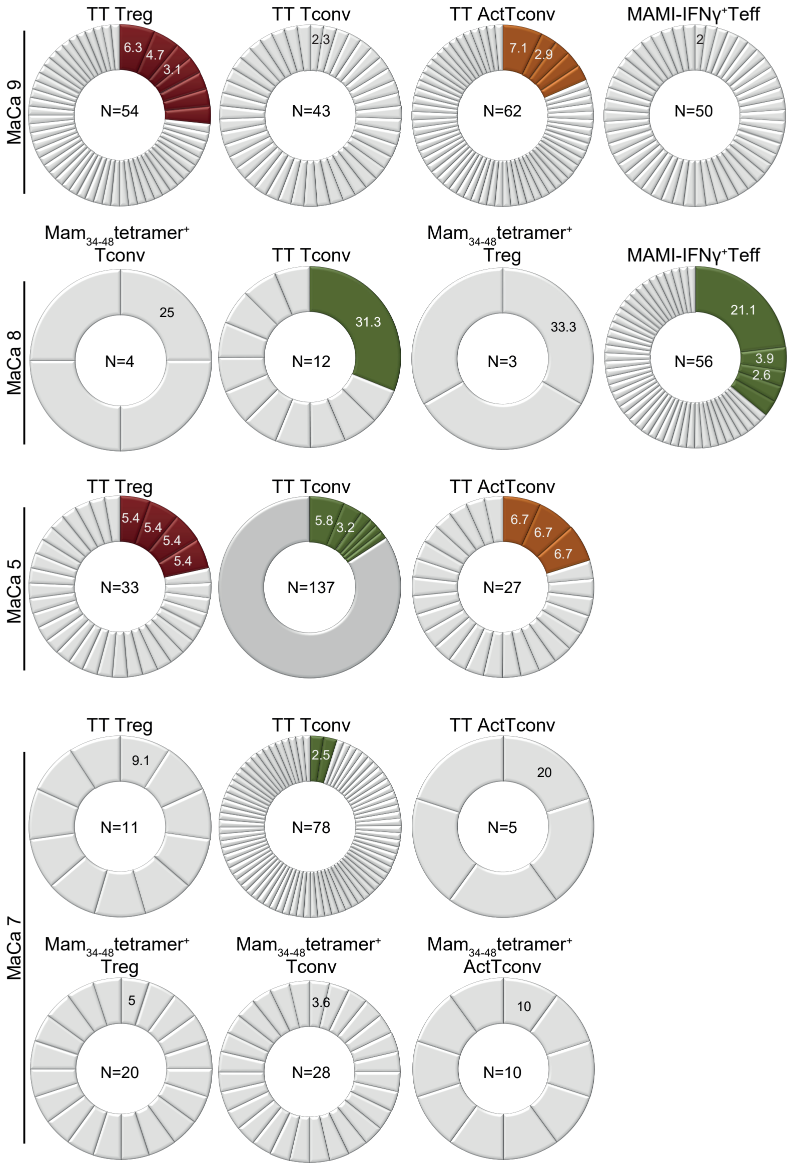

Supplementary Figure 7. TCRαβ clonal frequency distribution of circulating and tumor-infiltrating CD4⁺ TC in breast cancer patients. Pie chart frequency distribution of all single-cell clones (unique TCRαβ nucleotide sequences) obtained from tumor-tissue (TT) infiltrating TT Treg (red), TT Tconv (green), TT ActTconv (orange) and from blood-derived MAMI-IFNγ⁺Teff (green) but also mam$_{34-48}$-tetramer⁺ Tconv, Treg and ActTconv of breast cancer patients with mammary gland adenocarcinoma (MaCa), presented as one pie-slice per TCRαβ clone in order of decreasing percentage (%). TCRαβ sequences were characterized by single-cell sorting and TRACER-based prediction[5] after single-cell total transcriptome sequencing[6]. In contrast to colorful pie slices, white slices represent TCRαβ clones observed in only one cell and grey slices (MaCa 5, TT Tconv) represent the TCRαβ repertoire fraction that contains clones with frequency below 1%. N, total number of unique TCRαβ clones. n=4 for TT Tconv, TT Treg and TT ActTconv, n=2 for circulating MAMI-IFNγ⁺Teff and n=2 for mam$_{34-48}$-tetramer⁺ CD4⁺ TC. n refers to biologically independent replicates. Source data are provided as a Source Data file.

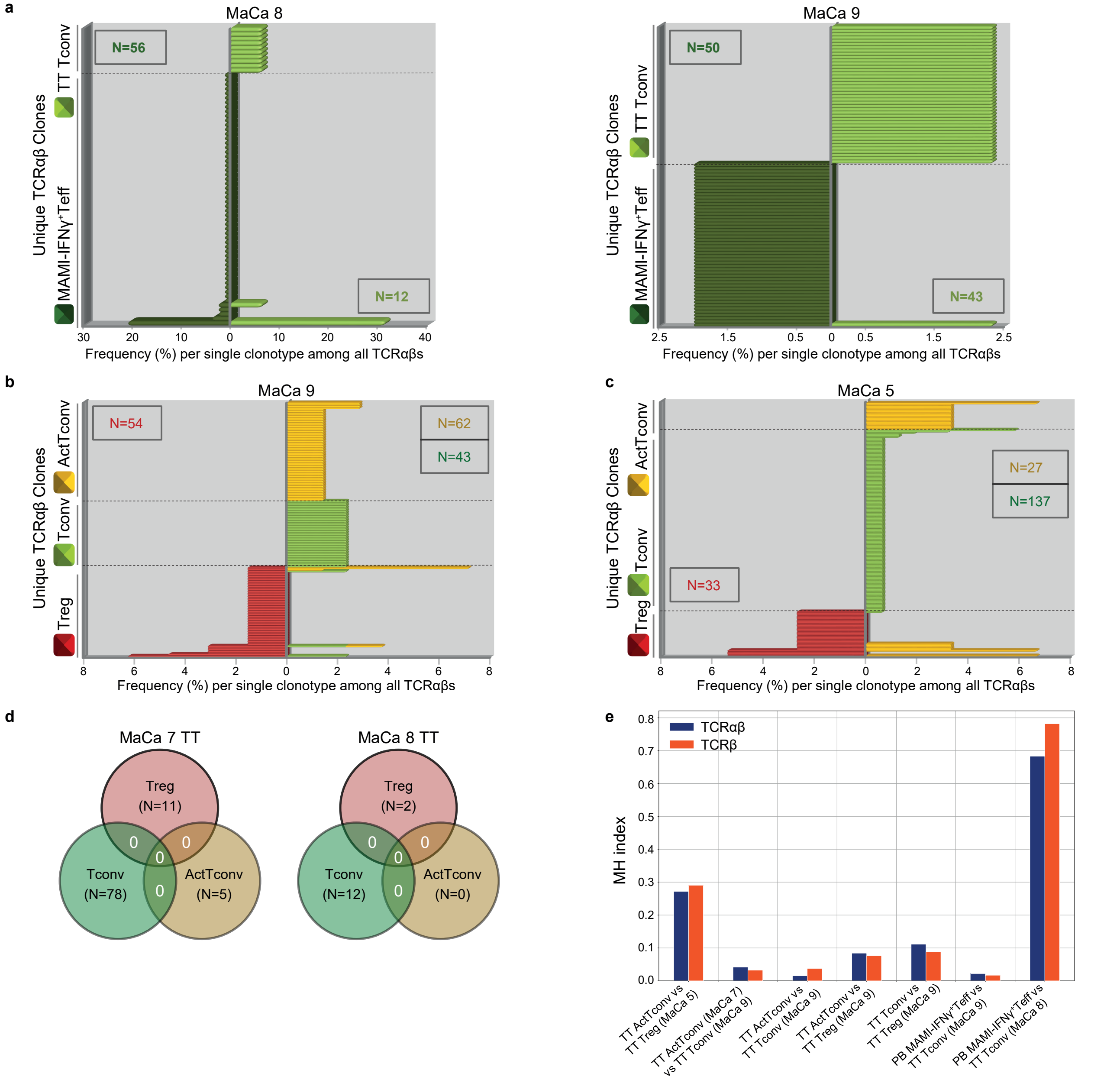

Supplementary Figure 8. TCRαβ overlap between blood-derived MAMI-reactive CD4⁺Teff and tumor-infiltrating Tconv Treg and ActTconv subsets in breast cancer patients. (**a, b, c**) Frequency of common TCRαβ clones (unique paired TCRαβ nucleotide sequences) shared (**a**) between circulating MAMI-IFNγ^{+}Teff and tumor-tissue (TT)-derived Tconv or (**b**, **c**) between intratumoral Tconv, Treg and ActTconv in relation to the frequencies of all TCRαβ clones detected per subset. Each bar represents a single clone (y axis) plotted against its frequency per subset (x axis). Clones are shown in order of decreasing (MAMI-IFNγ^{+}Teff, dark green; TT Treg, red) or increasing (TT Tconv, light green; TT ActTconv, orange) frequency. Clones of each subset are separated by a dashed black line. In case of overlapping clones between the two subsets (**a**) a dark green and a light green bar or (**b**, **c**) a red, a light green and an orange bar align on the y axis. N, total number of unique clones identified (**a**) among MAMI-IFNγ^{+}Teff (dark green) and TT Tconv (light green) or (**b**, **c**) among TT Tconv (light green), TT ActTconv (orange) and TT Treg (red). **(a)** n=2 and (**b**, **c**) n=4 biologically independent replicates. (**d**) Venn diagram showing the number of common (white) clones shared between tumor-infiltrating TT Tconv, Treg and ActTconv within the same patient. Data from 2 out of n=4 biologically independent replicates (**e**) Morisita-Horn (MH)-Indices of the observed similarity between circulating MAMI-IFNγ^{+}Teff and TT Tconv or between TT Tconv, Treg and ActTconv in MaCa patients. Similarity was calculated based on comparison of TCRβ clonotypes (unique TCRβ nucleotide sequences, red) or TCRαβ clones (unique paired TCRαβ nucleotide sequences, blue) between the compared subsets. The MH index is a unitless value that ranges between 0 for no similarity and 1 for complete overlap between two populations. n=4 biologically independent replicates. Source data are provided as a Source Data file.

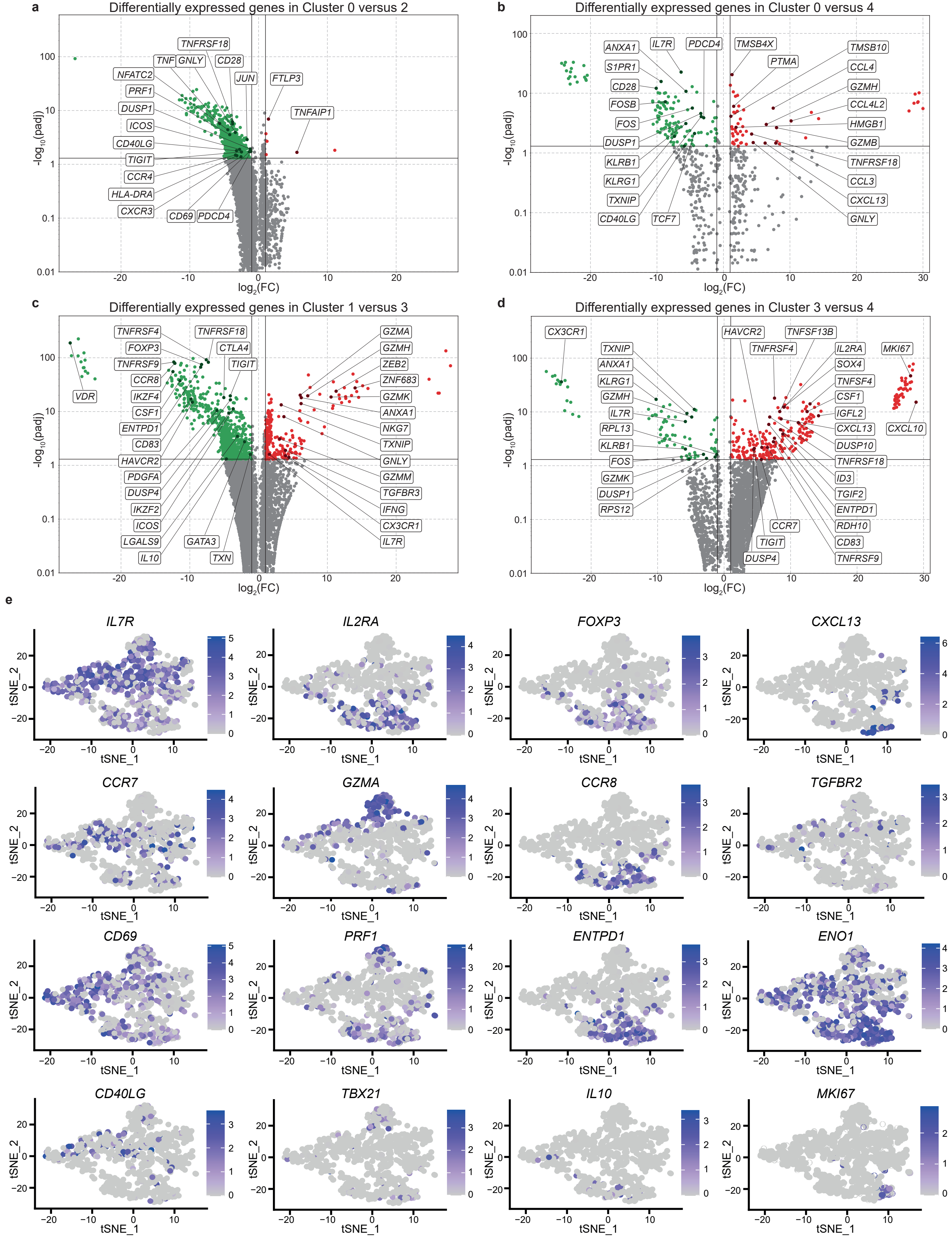

Supplementary Figure 9. Deseq2 pairwise comparison between different tumor-infiltrating CD4⁺ TC clusters. Volcano plots depicting genes differentially expressed between transcriptome clusters of tumor-infiltrating CD4⁺ TC from n=4 breast cancer patients. n, biologically independent replicates. Pairwise comparison between (**a**) cluster 0 (green) and cluster 2 (red), (**b**) cluster 0 (green) and cluster 4 (red), (**c**) cluster 1 (green) and cluster 3 (red) and (**d**) cluster 3 (green) and cluster 4 (red) using two-class differential expression analysis with the function DESeq with default parameters, which performs negative-binomial generalized linear model fitting using Wald tests for significance. FC, Fold Change. padj, adjusted *P* values. (**e**) t-SNE representation of tumor-infiltrating CD4⁺ TC showing expression of different marker genes of clusters 0, 1, 2, 3 and 4 ranging from 0 (grey) to maximum expression (blue).

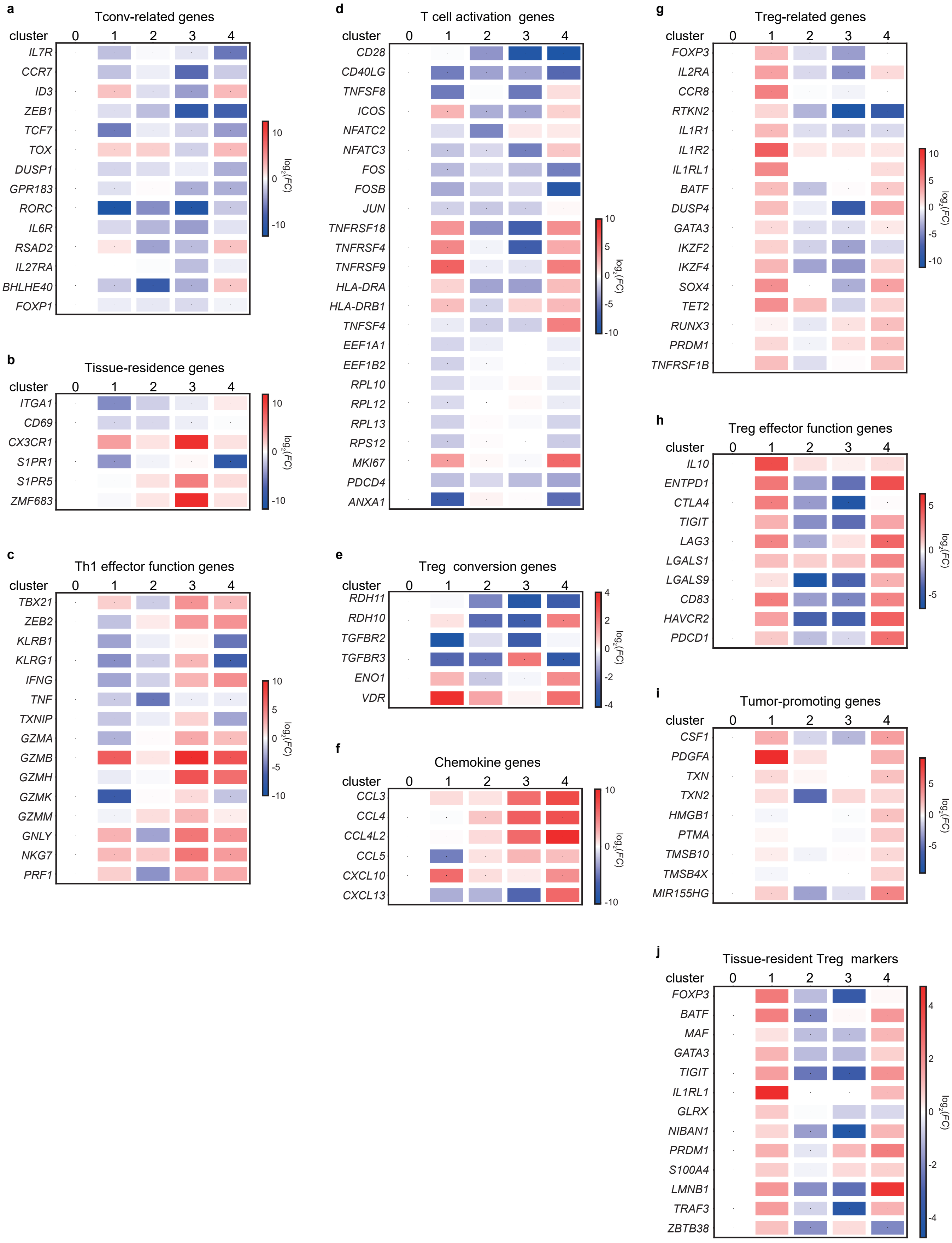

Supplementary Figure 10. Differential gene expression between distinct tumor-infiltrating CD4⁺ TC clusters. Heatmap representation of the $\log_2$(FC) of the expression levels of genes differentially expressed in transcriptome cluster 0, 1, 2, 3 and 4 versus the early activation cluster 0 from tumor-infiltrating CD4⁺ TC of 4 breast cancer patients. Genes are organized in block heatmaps according to their function: (**a**) Tconv-related genes, (**b**) Tissue-residence genes, (**c**) Th1 effector function genes, (**d**) TC activation genes, (**e**) Treg conversion genes, (**f**) chemokine genes, (**g**) Treg-related genes, (**h**) Treg effector function genes, (**i**) Tumor-promoting genes and (**j**) Tissue-resident Treg markers. FC, Fold Change.

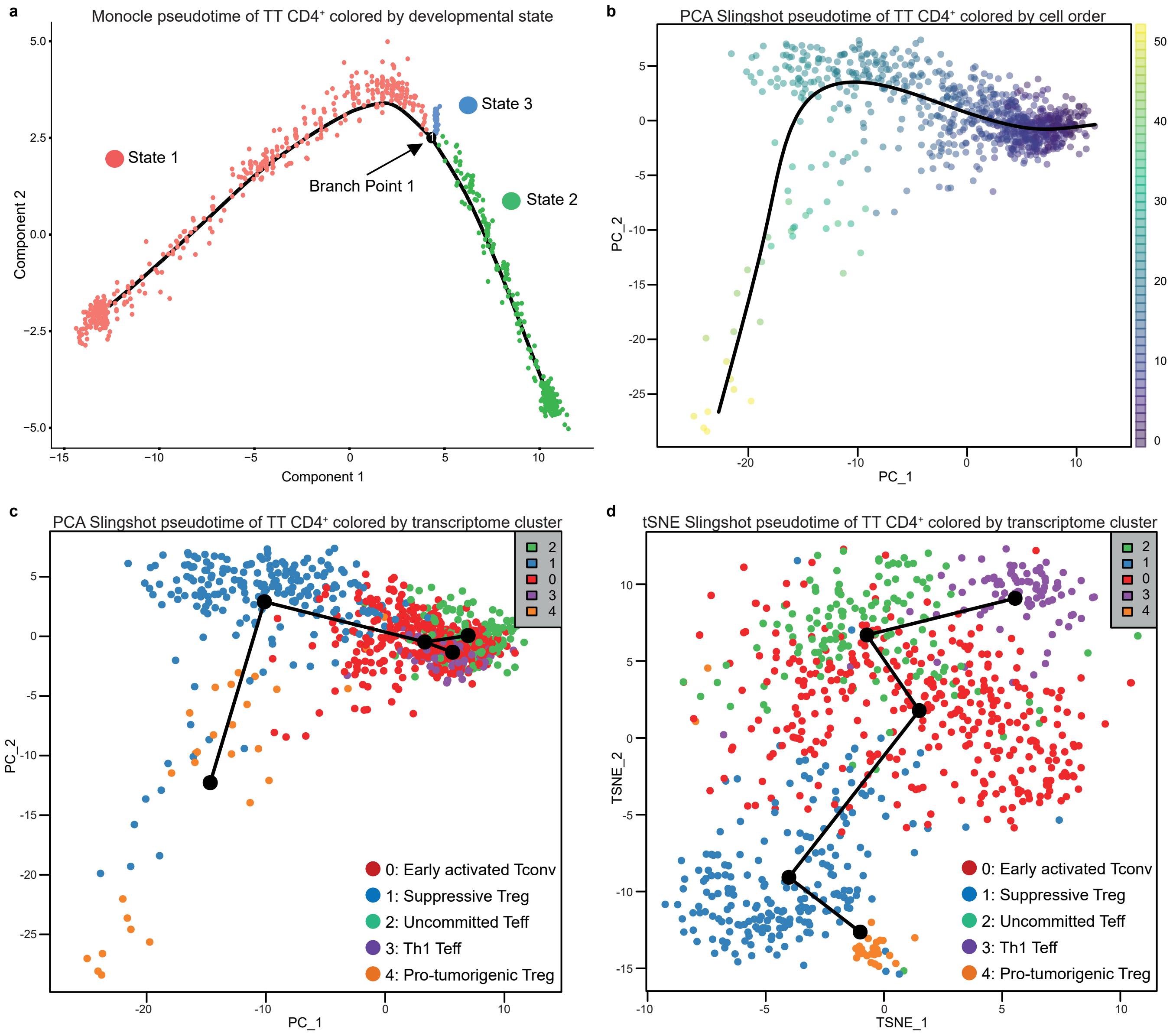

Supplementary Figure 11. Single-cell trajectory of tumor-infiltrating CD4⁺ TC from breast cancer patients. (**a**) Monocle pseudotime trajectory of tumor-tissue (TT) infiltrating CD4⁺ TC colored by developmental state. Monocle predicts one branch point (1) splitting into 3 distinct branches. Each branch represents 3 different developmental states. (**b, c, d**) Slingshot pseudotime trajectory of TT CD4⁺ TC colored (**b**) by cell order from time 0 (purple) towards more advanced states (yellow) or (**c**, **d**) by transcriptome cluster represented after (**b**, **c**) Principal Component Analysis (PCA) or (**d**) tSNE analysis. Cluster 0 is the central point of the trajectory developing towards cluster 1 and 4 or cluster 2 and 3. (**b-c**) Using PCA linear dimensional reduction the separation between cluster 2 and 3 is less clear showing cluster 0 differentiating separately either towards cluster 2 or towards cluster 3. (**d**) tSNE non-linear dimensional reduction generates efficient separation between cluster 2 and 3, suggesting the differentiation of cluster 0 into cluster 3 through cluster 2.

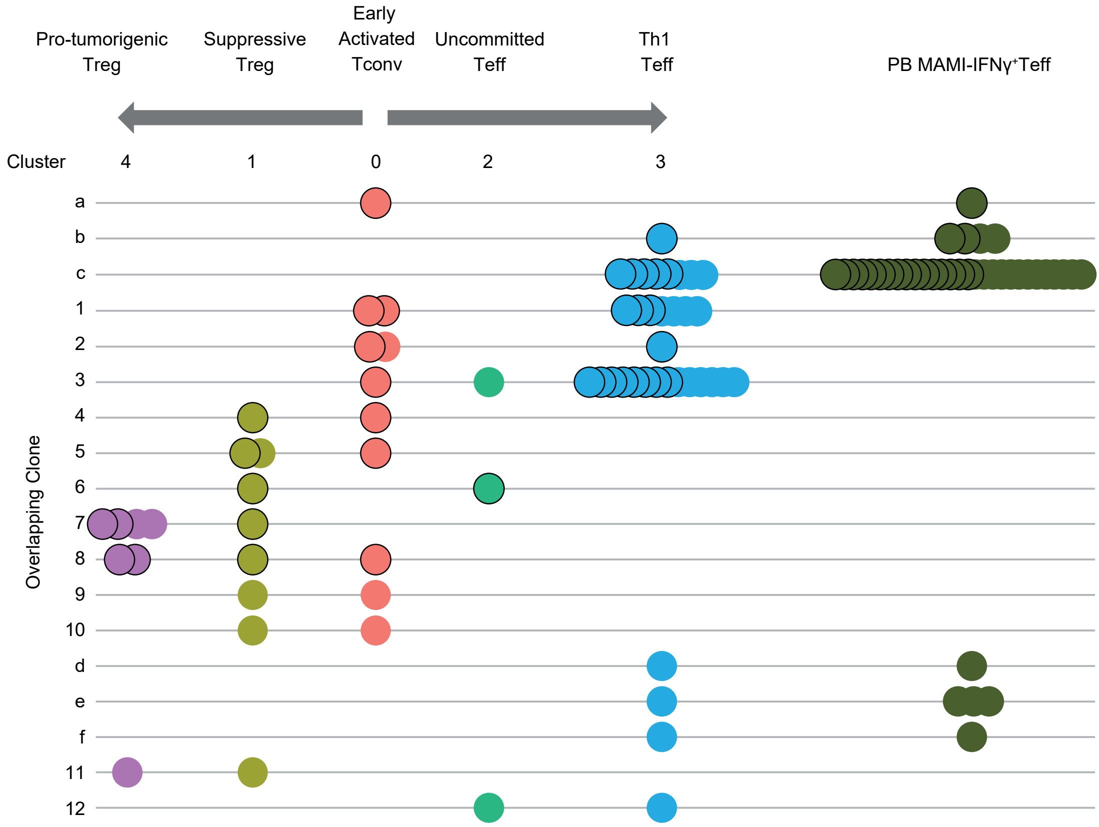

Supplementary Figure 12. Overlapping TCR clones between blood-derived MAMI-reactive IFNγ^{+}Teff and tumor-infiltrating CD4^{+} subsets in breast tumors. Each line represents a unique overlapping clone. Closed circles surrounded by a solid black line correspond to TCRαβ clones, while open circles represent TCRα or TCRβ clonotypes alone, as the second TCR chain nucleotide sequence could not be detected. MAMI-reactive IFNγ^{+}Teff from peripheral blood (PB) are depicted in dark-green, early activated Tconv cluster 0 in red, uncommitted Teff cluster 2 in green, Th1 Teff cluster 3 in light blue, suppressive Treg cluster 1 in dark orange and pro-tumorigenic Treg cluster 4 in purple.

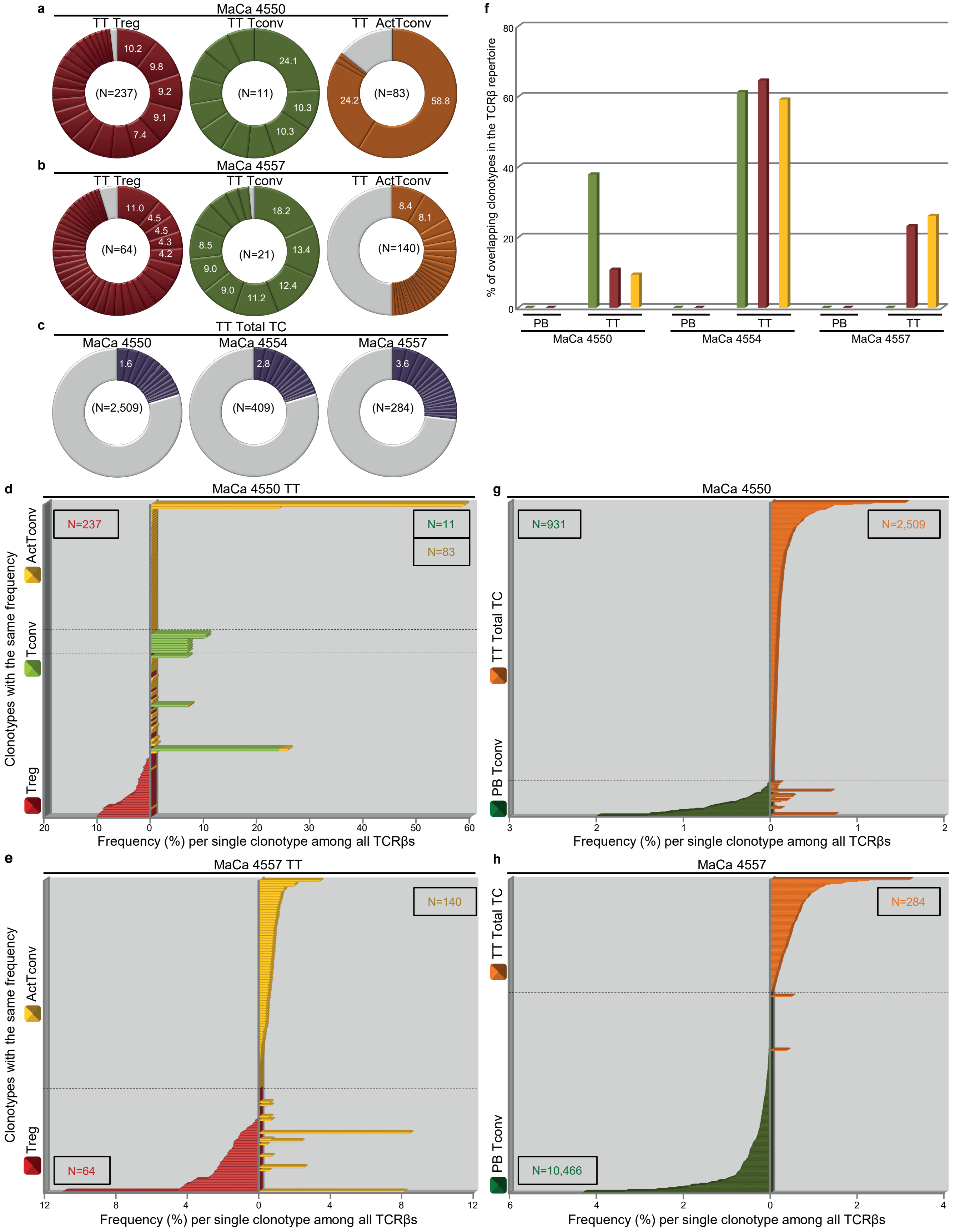

Supplementary Figure 13. TCRβ similarity between blood-derived Tconv and tumor-infiltrating subsets in breast tumors. (**a, b, c**) Pie chart representation of all clonotypes (unique TCRβ nucleotide sequences) recovered per tumor tissue (TT)-infiltrating subset in order of decreasing percentage (%, white) with the size of each colored pie-slice proportional to the frequency of the depicted clonotype. Grey slices represent the TCRβ repertoire fraction that contains clones with frequency below 1% (**a**, TT Treg and TT ActTconv; **b**, all subsets; **c**, all patients). TT Treg (red), TT Tconv (green) and TT ActTconv (orange) in breast cancer patient with mammary gland adenocarcinoma MaCa 4550 (**a**) and 4557 (**b**). (**c**) TT Total TC (blue) from 10x serial 25µm FFPE breast TT sections from n=3 biologically independent replicates. (**d, e, g, h**) The frequency of overlapping clonotypes between (**d**, **e**) TT Treg and TT Tconv and/or TT ActTconv or (**g**, **h**) between peripheral blood (PB)-derived Tconv and tumor-infiltrating TT Total TC in relation to the frequency distribution of all clonotypes within each subset in patient (**d**, **g**) MaCa 4550 and (**e**, **h**) 4557. Each bar represents a group of clonotypes with identical

frequencies (y axis) plotted against the frequency of each clonotype per group (x axis) in order of decreasing (TT Treg, red; PB Tconv, dark green) and increasing (TT Tconv, green; TT ActTconv, orange; TT Total TC, brown) frequency. Clones of each subset are separated by a dashed black line. In case of overlapping clones between the subsets (**d**, **e**) a red, a green and an orange bar or (**g**, **h**) a dark green and a brown bar align on the y axis. (**f**) Total frequency of Treg clonotypes overlapping with Tconv or ActTconv among Tconv (green), Treg (red) or ActTconv (orange) either in PB or within TT in n=3 MaCa patients. MaCa 4550 data obtained from Treg and Tconv in PB were excluded, as the observed overlap between the two subsets could be explained by background contamination during sorting, as explained in the main text of the manuscript. N, total number of clonotypes recovered per population. MaCa: n=3, n refers to biologically independent replicates. Source data are provided as a Source Data file.

Supplementary Table 1. Purity of sorted Treg and Tconv populations

Tested individual	Sorted subset	[a] Treg proportion (%)	[a] Tconv proportion (%)	[b] Treg Contamination 95%-Confidence Interval	[b] Tconv Contamination 95%- Confidence Interval
MaCa 4220	Tconv	0.2	99.8	0.01 - 0.95	
MaCa 4220	Treg	99.8	0.2		0.01 - 1.10
MaCa 4223	Tconv	0.2	99.8	0.01 - 0.95	
MaCa 4223	Treg	99.8	0.2		0.01 - 1.10
MaCa 4572	Tconv	0.1	99.9	0.03 - 0.24	
MaCa 4572	Treg	98.1	1.9		1.15 - 2.94
MaCa 4573	Tconv	0.1	99.9	0.03 - 0.24	
MaCa 4573	Treg	98.1	1.9		1.15 - 2.94
MaCa 4578	Tconv	0.1	99.9	0.03 - 0.24	
MaCa 4578	Treg	98.1	1.9		1.15 - 2.94
MaCa 4550	Tconv	0	100	0 - 1.32	
MaCa 4550	Treg	98.3	1.7		0.46 - 4.32
MaCa 4554	Tconv	0	100	0 - 1.32	
MaCa 4554	Treg	98.3	1.7		0.46 - 4.32
MaCa 4557	Tconv	0	100	0 - 1.32	
MaCa 4557	Treg	98.3	1.7		0.46 - 4.32
HD1	Tconv	0.2	99.8	0.05 – 0.52	
HD1	Treg	97.2	2.8		1.58 - 4.56
HD2	Tconv	0.1	99.9	0.07 – 0.13	
HD2	Treg	97.9	2.1		1.97 – 2.23
HD3	Tconv	0	100	0 – 0.07	
HD3	Treg	98	2		0.96 – 3.66

MaCa, breast cancer patient with mammary gland adenocarcinoma; HD, Healthy Donor; Treg proportion (%), the frequency of $CD25^{+}CD127^{-/low}$ cells measured among sorted Tconv/Treg; Tconv proportion (%), the proportion of $CD25^{-}CD127^{+/-}$ cells measured among sorted Treg/Tconv. n=11 biological replicates including 8 MaCa and 3 HD tested in 6 independent experiments.

[a] Purity of FACS-sorted Treg and Tconv from peripheral blood of MaCa patients and HD, as measured at the beginning of the sorting, after reanalysis of a small number of sorted Treg and Tconv, respectively, using the same gating strategy, settings and FACS sorter as during sorting.

[b] The expected contamination of the complete sorted population from the other subset, as calculated with exact two-sided 95%-Pearson-Clopper Confidence Interval.

Supplementary Table 2. Characteristics of sorted TC subsets and recovered TCRβ sequences

Tested individual sorted subset	Sorted Cell Number	MAMI-stimulated Tconv Cell number	IgG-stimulated Tconv Cell Number	MAMI-IFNγ$^+$Teff among Tconv	IgG-IFNγ$^+$Teff among Tconv	MAMI-IFNγ$^+$Teff Cell Number	IgG-IFNγ$^+$Teff Cell Number	MAMI-IFNγ$^+$Teff φ_{max}	IgG-IFNγ$^+$Teff φ_{max}
MaCa 4220 Tconv	$29.5x10^{5}$	$20x10^{5}$	$5x10^{5}$	0.2%	0.1%	48	n.t	6.1%	n.t
MaCa 4223 Tconv	$6.25x10^{5}$	$5x10^{5}$	10^{5}	0.1%	0%	40	n.t	7.2%	n.t
MaCa 4572 Tconv	$5.5\ x10^{5}$	$2.4x10^{5}$	$2.4x10^{5}$	0.7%	0.5%	88	88	3.3%	3.3%
MaCa 4573 Tconv	$4\ x10^{5}$	$1.8x10^{5}$	$1.8x10^{5}$	2.4%	1.5%	200	300	1.5%	1.0%
MaCa 4578 Tconv	$6\ x10^{5}$	$2.2\ x10^{5}$	$1.1x10^{5}$	1.6%	1.6%	400	352	0.7%	0.8%

Tested individual sorted subset	Sorted Cell Number	Total RNA (ng)	Sequenced RNA (ng)	Total TCRβ sequences	TCRβ clonotypes	φ_{max}
MaCa 4220 Treg	$4.4x10^{4}$	90.8	90.8	5,443	1,645	0.01%
MaCa 4223 Treg	$1.6x10^{4}$	29.2	29.2	3,379	214	0.02%
MaCa 4572 Treg	$1.3x10^{4}$	51.6	51.6	4,789	521	0.02%
MaCa 4573 Treg	$1.5x10^{4}$	37.2	37.2	4,929	474	0.02%
MaCa 4578 Treg	$6.7x10^{4}$	52.8	52.8	2,942	933	0.004%
MaCa 4550 Tconv	$2x10^{5}$	96	96	2,747	931	0.001%
MaCa 4550 Treg	$8x10^{3}$	28.8	28.8	5,580	303	0.04%
MaCa 4554 Tconv	$6.5x10^{6}$	2,040	980	111,766	8,294	0.0001%
MaCa 4554 Treg	$4.5x10^{5}$	96	48	277,467	4,359	0.001%
MaCa 4557 Tconv	$5.8x10^{6}$	1,809	869	155,146	10,466	0.0001%
MaCa 4557 Treg	$3x10^{5}$	135.6	67.8	258,245	4,472	0.002%
HD1 Tconv	$8.5x10^{7}$	8,710	2,487	195,977	16,745	0.00001%
HD1 Treg	$1.5x10^{6}$	1,139	845.1	1,487	1,107	0.0003%
HD2 Tconv	$4.85x10^{7}$	21,550	18,060	173,881	34,994	0.000007%
HD2 Treg	$2.15x10^{6}$	1,329	1,038	2,097	1,710	0.0002%
HD3 Tconv	$5x10^{7}$	28,560	19,871	215,133	49,710	0.00009%
HD3 Treg	$1.25x10^{6}$	762	762	2,437	1,737	0.0002%

MaCa, breast cancer patient with mammary gland adenocarcinoma; HD, Healthy Donor; MAMI, Mammaglobin; IgG; human Immunoglobulin; n.t, not tested; TCRβ sequence characterization among MAMI- and IgG-reactive IFNγ$^+$Teff versus total Treg from peripheral blood of five MaCa patients or among total Tconv versus total Treg from peripheral blood of three additional MaCa patients and three HDs tested in six independent experiments. For each Tconv and Treg subset the number of sorted cells after sorting is indicated together with the respective amount of extracted RNA and the quantity of RNA used for high-throughput sequencing[2,3,4]. For some patients the absolute number of total Tconv obtained by FACS sorting is shown in combination with the Tconv cell number activated either with MAMI- or with IgG-pulsed DC. The consequent frequency of MAMI- and IgG-reactive IFNγ$^+$Teff among Tconv follows together with the number of cells analyzed for their TCRβ CDR3 sequence either by single-cell sorting and single-cell multiplex RT-PCR[1] (MaCa 4220, 4223 and 4272) or by FACS sorting and TCRβ high-throughput sequencing[2,3,4] of cells directly sorted into Reverse Transcription buffer (MaCa 4573 and 4578). The total number of all in-frame TCRβ nucleotide sequences recovered per subset is depicted together with the number of all clonotypes (unique TCRβ nucleotide sequences). The maximum frequency (%) of an unobserved clone with 95% Confidence Interval is indicated by φ_{max}[7].

Supplementary Table 3. TCRβ similarity between TC subsets in peripheral blood and tumor

Peripheral Blood	HD1	HD2	HD3	MaCa 4220	MaCa 4223	MaCa 4572	MaCa 4573	MaCa 4578	MaCa 4550	MaCa 4554	MaCa 4557
MAMI- vs IgG-IFNγ$^+$Teff	n.t	n.t	n.t	n.t	n.t	0	0.0006	0.03	n.t	n.t	n.t
MAMI-IFNγ$^+$Teff vs Treg	n.t	n.t	n.t	0	0	0	0	0.0003	n.t	n.t	n.t
IgG-IFNγ$^+$Teff vs Treg	n.t	n.t	n.t	n.t	n.t	0	0.00006	0.00001	n.t	n.t	n.t
Tconv vs Treg	0.00002	0.0006	0.0001	n.t	n.t	n.t	n.t	n.t	0.023	0	0
**Tumor Tissue**	**HD1**	**HD2**	**HD3**	**MaCa 4220**	**MaCa 4223**	**MaCa 4572**	**MaCa 4573**	**MaCa 4578**	**MaCa 4550**	**MaCa 4554**	**MaCa 4557**
TT Tconv vs TT Treg	n.a	n.a	n.a	n.t	n.t	n.t	n.t	n.t	0.0008	0.75839	0
TT ActTconv vs TT Treg	n.a	n.a	n.a	n.t	n.t	n.t	n.t	n.t	0.0006	0.75481	0.38242
TT Tconv vs TT ActTconv	n.a	n.a	n.a	n.t	n.t	n.t	n.t	n.t	0.015	0.73439	0
**Peripheral Blood vs Tumor Tissue**	**HD1**	**HD2**	**HD3**	**MaCa 4220**	**MaCa 4223**	**MaCa 4572**	**MaCa 4573**	**MaCa 4578**	**MaCa 4550**	**MaCa 4554**	**MaCa 4557**
PB Tconv vs TT Tconv	n.a	n.a	n.a	n.t	n.t	n.t	n.t	n.t	0	0	0
PB Tconv vs TT Treg	n.a	n.a	n.a	n.t	n.t	n.t	n.t	n.t	0	0	0
PB Tconv vs TT ActTconv	n.a	n.a	n.a	n.t	n.t	n.t	n.t	n.t	0	0	0
PB Tconv vs TT Total TC	n.a	n.a	n.a	n.t	n.t	n.t	n.t	n.t	0.053	0	0.0002
PB Treg vs TT Tconv	n.a	n.a	n.a	n.t	n.t	n.t	n.t	n.t	0	0	0
PB Treg vs TT Treg	n.a	n.a	n.a	n.t	n.t	n.t	n.t	n.t	0	0	0
PB Treg vs TT ActTconv	n.a	n.a	n.a	n.t	n.t	n.t	n.t	n.t	0	0	0
PB Treg vs TT Total TC	n.a	n.a	n.a	n.t	n.t	n.t	n.t	n.t	0	0	0

HD, Healthy Donor; MaCa, breast cancer patient with mammary gland adenocarcinoma; MAMI, Mammaglobin; IgG, human Immunoglobulin; vs, versus; PB, Peripheral Blood; TT, Tumor Tissue; n.t, not tested; n.a, not applicable; Morisita-Horn (MH)-Indices of the observed similarity between Treg and Tconv subsets in peripheral blood (n=11) and tumor tissue (n=3) of 8 MaCa patients and 3 HDs. n refers to biologically independent replicates. Similarity was calculated based on comparison of clonotypes (unique TCRβ nucleotide sequences) between the compared subsets. The MH index is a unitless value that ranges between 0 for no similarity and 1 for complete overlap between two populations, here depicted in yellow for $0 \leq MH \leq 0.1$, orange for $0.1 < MH \leq 0.5$ and brown for $MH > 0.5$. In PB from MaCa patients the total Treg population is compared against the total Tconv population or against the MAMI- or the IgG-reactive IFNγ$^+$Teff subset. In breast tumors the TT Treg population is analyzed against the TT Tconv or the TT ActTconv isolated within the same FFPE tissue section. Last, within each MaCa patient, PB Tconv and PB Treg are compared against TT Tconv, TT Treg, TT ActTconv but also against TT total TC obtained from 10 serial neighboring 25µm FFPE tissue sections from the same tumor. n refers to biological replicates tested in 6 (PB) and 3 (TT) independent experiments, respectively.

Supplementary Table 4. Characteristics of tumor-infiltrating TC subsets and recovered TCRβ sequences

Tested individual subset	Laser-microdissected Cell Number	Total TCRβ sequences	TCRβ clonotypes	φ_{max}
MaCa 4550 TT Tconv	863	7,280	11	0.35%
MaCa 4550 TT Treg	1,239	218,161	237	0.24%
MaCa 4550 TT ActTconv	1,030	2,202	83	0.29%
MaCa 4550 TT Total TC	n.a	449,270	2,509	n.a
MaCa 4554 TT Tconv	1,006	10,281	39	0.30%
MaCa 4554 TT Treg	1,047	24,751	82	0.29%
MaCa 4554 TT ActTconv	1,172	14,833	42	0.26%
MaCa 4554 TT Total TC	n.a	3,572	409	n.a
MaCa 4557 TT Tconv	1,003	34,341	21	0.30%
MaCa 4557 TT Treg	1,210	54,181	64	0.25%
MaCa 4557 TT ActTconv	1,920	93,949	140	0.16%
MaCa 4557 TT Total TC	n.a	27,956	284	n.a

MaCa, breast cancer patient with mammary gland adenocarcinoma; TT, tumor tissue; TC, T cells; n.a, not applicable; TCRβ sequence characterization of single-cell laser-microdissected TT Tconv, TT Treg and TT ActTconv isolated within the same FFPE TT sections and of TT Total TC from 10 additional serial 25μm FFPE TT sections within the same breast tumor after gDNA-based high-throughput TCRβ sequencing. The number of single-cells laser-microdissected per subset is shown, followed by the total number of recovered TCRβ nucleotide sequences together with the respective number of in-frame clonotypes (unique TCRβ nucleotide sequences). The maximum frequency (%) of an unobserved clone with 95% Confidence Interval is indicated by φ_{max}[7]. n=3 biological replicates performed in 3 independent experiments.

Supplementary Table 5. Primers for TCRβ transcript amplification

**Forward primers**		
**Primer name**	**forward primer sequence (5‘-3‘)**	**2nd PCR reaction**
TRBV9/TRBV5	ACAGCAAGTGAC<TAG>CTGAGATGCTC	A
TRBV25	GATCACTCTGGAATGTTCTCAAACC	A
TRBV10	CCAAGACACAAGGTCACAGAGACA	A
TRBV20	GAGTGCCGTTCCCTGGACTTTCAG	B
TRBV28	GTAACCCAGAGCTCGAGATATCTA	B
TRBV2	GGTCACACAGATGGGACAGGAAGT	B
TRBV29	TCCAGTGTCAAGTCGATAGCCAAGTC	C
TRBV7	ATGTAACT<CT>TCAGGTGTGATCCAA	C
TRBV27	GTGACCCAGAACCCAAGATACCTC	C
TRBV7	GTGTGATCCAATTTCAGGTCATAC	D
TRBV12	GGTGACAGAGATGGGACAAGAAGT	D
TRBV11	CAGTCTCCCAGATATAAGATTATAGAG	D
TRBV19	CACTCAGTCCCCAAAGTACCTGTT	E
TRBV30	GTCAGATCTCAGACTATTCATCAATGG	E
TRBV4	TACGCAGACACCAA<GA>ACACCTGGTCA	E
TRBV3	CCCAGACTCCAAAATACCTGGTCA	E
TRBV18	TGCAGAACCCAAGACACCTGGTCA	E
TRBV21	AAGGTCACCCAGAGACCTAGACTT	F
TRBV14	ATAGAAGCTGGAGTTACTCAGTTC	F
TRBV23	ACAAAGATGGATTGTACCCCCGAA	F
TRBV6	GTGTCACTCAGACCCCAAAATTCC	G
TRBV24	GTTACCCAGACCCCAAGGAATAGG	G
TRBV13	CTGATCAAAGAAAAGAGGGAAACAGCC	H
TRBV15	CAAGATACCAGGTTACCCAGTTTG	H
**Reverse primers**		
**TRBV gene names**	**forward primer sequence (5-3)**	**PCR reaction**
BCRT	CACCAGTGTGGCCTTTTG	Reverse Transcription
3 BCRT	GCTTCTGATGGCTCAAACACAGC	1st PCR
5BCRT	GGAACACGTTTTTCAGGTCCT	2nd and 3rd PCR

The nucleotide sequence of the primers used for the three-step single-cell RT-PCR applied for the amplification of the TCRβ chain CDR3 region[1]. A-H correspond to the eight different groups of pooled primers used per reaction in the 2nd multiplex PCR step.

References

1. Tanaka-Harada, Y. *et al*. Biased usage of BV gene families of T-cell receptors of WT1 (Wilms' tumor gene)-specific CD8+ T cells in patients with myeloid malignancies. *Cancer Sci.* **101**(3), 594-600 (2010).
2. Ruggiero, E. *et al*. High-resolution analysis of the human T cell receptor repertoire. *Nat Commun* **6**, 8081, doi: 10.1038/ncomms9081 (2015).
3. Schmidt, M. *et al*. High-resolution insertion-site analysis by linear amplification-mediated PCR (LAM-PCR). *Nat. Methods* **4**, 1051–1057 (2007).
4. Paruzynski, A. *et al*. Genome-wide high-throughput integrome analyses by nrLAM-PCR and next-generation sequencing. *Nat. Protoc*. **5**, 1379–1395 (2010).
5. Stubbington, M.J.T. *et al*. T cell fate and clonality inference from single-cell transcriptomes. *Nat Methods* **13**(4), 329-332 (2016).
6. Macaulay, I.C. *et al*. Separation and parallel sequencing of the genomes and transcriptomes of single cells using G&T-seq. *Nature Protocols* **11**, 2081–2103 (2016).
7. Baron, V. *et al*. The repertoires of circulating human CD8(+) central and effector memory T cell subsets are largely distinct. *Immunity* **18**(2), 193-204 (2003).
