# Supplementary material for: Common clonal origin of conventional T cells and induced regulatory T cells in breast cancer patients
Source: Nat Commun. 2021 Feb 18;12:1119. doi: 10.1038/s41467-021-21297-y (PMC7893042; doi:10.1038/s41467-021-21297-y)
Supplement: Supplementary file 2 — Description of Additional Supplementary Files [file 41467_2021_21297_MOESM2_ESM.pdf]

Description of Additional Supplementary Files

File Name: Supplementary Software 1
Description: **Quality Control and patient-specific single-cell RNA sequencing data analysis.** R code for single-cell RNA sequencing data analysis. Step 1, Quality Control based on library size and number of expressed genes. Step 2, Transcriptome cluster analysis and comparison between patients and cell types. Step 3, Marker gene analysis per transcriptome cluster and cell type.

File Name: Supplementary Software 2
Description: **Pseudotime analysis of single-cell RNA sequencing data.** The code contains pseudotime analysis and cell timing order using two different methods. Method 1 describes Monocle pseudotime analysis and Method 2 describes Slingshot pseudotime analysis of single-cell RNA sequencing data.
